# Receptor architecture of macaque and human early visual areas: not equal, but comparable

**DOI:** 10.1007/s00429-021-02437-y

**Published:** 2021-12-20

**Authors:** Lucija Rapan, Meiqi Niu, Ling Zhao, Thomas Funck, Katrin Amunts, Karl Zilles, Nicola Palomero-Gallagher

**Affiliations:** 1 Institute of Neuroscience and Medicine (INM-1), Research Centre Jülich, 52425 Jülich, Germany; 2 C. & O. Vogt Institute for Brain Research, Heinrich-Heine-University, 40225 Düsseldorf, Germany; 3 Department of Psychiatry, Psychotherapy, and Psychosomatics, Medical Faculty, RWTH Aachen, Aachen, Germany

**Keywords:** Brain mapping, Cytoarchitecture, Comparative analysis, Visual processing, Homology

## Abstract

Existing cytoarchitectonic maps of the human and macaque posterior occipital cortex differ in the number of areas they display, thus hampering identification of homolog structures. We applied quantitative in vitro receptor autoradiography to characterize the receptor architecture of the primary visual and early extrastriate cortex in macaque and human brains, using previously published cytoarchitectonic criteria as starting point of our analysis. We identified 8 receptor architectonically distinct areas in the macaque brain (mV1d, mV1v, mV2d, mV2v, mV3d, mV3v, mV3A, mV4v), and their respective counterpart areas in the human brain (hV1d, hV1v, hV2d, hV2v, hV3d, hV3v, hV3A, hV4v). Mean densities of 14 neurotransmitter receptors were quantified in each area, and ensuing receptor fingerprints used for multivariate analyses. The 1st principal component segregated macaque and human early visual areas differ. However, the 2nd principal component showed that within each species, area-specific differences in receptor fingerprints were associated with the hierarchical processing level of each area. Subdivisions of V2 and V3 were found to cluster together in both species and were segregated from subdivisions of V1 and from V4v. Thus, comparative studies like this provide valuable architectonic insights into how differences in underlying microstructure impact evolutionary changes in functional processing of the primate brain and, at the same time, provide strong arguments for use of macaque monkey brain as a suitable animal model for translational studies.

**Supplementary Information:**

The online version contains supplementary material available at 10.1007/s00429-021-02437-y.

## Introduction

The visual modality is possibly the most developed in the primate brain, and occupies the largest amount of cerebral cortex (Van Essen 2003). In primates, the early visual cortex also provides an ideal model for understanding the entire visual system in general, because of the hierarchical progression in its structural and functional organization. Within the primary visual cortex (V1), optic fibers carrying information from the lower and upper visual fields terminate on the dorsal and ventral banks of the *calcarine sulcus*, respectively (Gillen 2015), and information provided by this segregation is carried on to higher visual areas, which were, therefore, categorized as belonging to one of two major visual streams (Ungerleider 1982), i.e., dorsal (occipitoparietal) and ventral (occipitotemporal) streams. Overall, the simple definition of the original dorsal/ventral dichotomy resulted in a too restraining idea of a spatial and object-related perception of the dorsal and ventral flow, and it has therefore been suggested that visual information is conveyed between two systems at multiple stages and locations along the processing way. Hierarchical organization of the visual processing would be composed of multiple, intertwined processing streams, which, at a lower level, are related to the compartmental organization of early visual areas (V1–V3) and, at a higher level, are associated with the distinction between processing centers of the parietal and temporal cortex (Cloutman 2013; Felleman and Van Essen 1991).

Comparative studies of the human and macaque visual system showed that the early visual areas V1, V2 and V3 are located more posterior and medially in humans than the correspondingly marked areas in macaques. This is particularly true for V1, which in humans is located almost entirely in the *cas*, whereby macaque V1 occupies a substantial portion of the operculum on the lateral surface of the occipital lobe (Orban et al. 2004; Schira et al. 2012). However, early and mid-level visual areas have also been shown to be evolutionarily well preserved, and to share a similar retinotopic organization, as well as basic functional traits across the primate species (Denys et al. 2004; Orban et al. 2003, 2004; Vanduffel et al. 2002). Although the receptor architecture of areas V1–V3, including their dorsal and ventral subdivisions, as well as of adjoining areas V3A dorsally and V4 ventrally has been comprehensively characterized in the human brain (Eickhoff et al. 2008, 2007) and these early visual areas were also part of a study on the organizational principles of the human brain as revealed by regional and laminar differences in receptor densities (Zilles and Palomero-Gallagher 2017b), the macaque monkey visual cortex has not yet been subject of such detailed receptor architectonic analyses, since existing studies concentrated mainly on macaque areas V1 and V2, analyzed only a single sample, examined a few receptor types, mostly from a single neurotransmitter system, or did not provide quantitative data (e.g., Hendry et al. 1990; Kötter et al. 2001; Rakic et al. 1988; Rakic and Lidow 1995; Rosier et al. 1993, 1991; Zilles and Clarke 1997; Zilles and Palomero-Gallagher 2017a).

Since transmitter receptors are key molecules of signal processing in the nervous system and determine the excitatory or inhibitory effect of neurotransmitters, they are a crucial prerequisite for understanding functional neuroanatomy. Neurotransmitter receptors are heterogeneously distributed throughout the cortex, and differences in receptor densities not only reveal cortical borders, but also segregate brain regions belonging to different cortical types (i.e., allocortex vs. isocortex) and functional systems (primary motor, somatosensory, visual, or auditory; language related vs. non-language related), and also identify hierarchical processing levels within a given functional system (Palomero-Gallagher and Zilles 2019; Zilles et al. 2015a; Zilles and Palomero-Gallagher 2017b). Furthermore, receptor autographic studies have also been shown to provide valuable insights into putative homologies between areas of the human and macaque monkey brain (Impieri et al. 2018; Niu et al. 2021; Palomero-Gallagher et al. 2013; Rapan et al. 2021).

Aim of the present study is to characterize transmitter expression in the primary visual cortex and in early extrastriate visual areas of the macaque brain and compare them to those of the human brain to identify the molecular basis of the systemic coherence of visual areas and provide a more comprehensive insight into the evolutionary aspect of the functional organization of the visual system in primates. Specifically, we addressed the following questions: (a) does the multi-receptor architecture of early visual areas reveal dorso-ventral differences in the non-human primate cortex, in the same manner as it does in humans (Eickhoff et al. 2008); and (b) do receptor fingerprints facilitate identification of similarities and differences between the macaque and human early visual areas?

## Materials and methods

### Subjects

We examined three adult male macaque monkey brains (*Macaca fascicularis*; brains ID: 11530, 11539, 11543; 6 ± 1 years of age; obtained from Covance Laboratories, Münster, Germany) for a combined cyto- and receptor architectonic analysis. Monkeys were killed by a lethal intravenous injection of sodium pentobarbital and brains were immediately extracted together with meninges and blood vessels to preserve cortical layer I. The procedures used in this study had the approval of the Institutional Animal Care and Use Committee, were carried out in accordance with the European and local Committees, and complied with the European Communities Council Directive 2010/63/EU.

Further, we used a total of five post-mortem human brains from donors (76 ± 3 years of age; 3 males) without a history of neurological or psychiatric diseases and obtained through the body donor program of the Department of Anatomy, University of Düsseldorf, Germany. Causes of death were sudden cardiac failure, multiorgan failure caused by sepsis and pneumonia, and lung edema.

### Tissue processing

The macaque brains were divided into left and right hemispheres (including cerebellum with brainstem) and further separated into an anterior and a posterior slab at the height of the most caudal part of the central sulcus. Human brains were removed at autopsy and divided into left and right hemispheres. Each hemisphere was then cut into slabs of approximately 3 cm each. All slabs were shock frozen in *N*-methylbutane (isopentane) at − 40 °C for 10–15 min, and serially sectioned (thickness 20 µm) in the coronal plane with a cryotome at − 20 °C, thaw-mounted on gelatin-coated glass slides, air dried and stored overnight at − 20 °C.

To examine the laminar and regional distribution patterns of 14 receptor types belonging to the classical neurotransmitters glutamate (AMPA, kainate and NMDA), GABA (GABA_A_, GABA_A_/BZ and GABA_B_), acetylcholine (muscarinic M_1_, M_2_ and M_3_), noradrenaline (α_1_ and α_2_), serotonin (5-HT_1A_ and 5-HT_2_) and dopamine (D_1_), and to enable comparison with cytoarchitectonic features, alternating sections were processed for quantitative in vitro receptor autoradiography according to previously published protocols (Palomero-Gallagher and Zilles 2018; Zilles et al. 2002), or for visualization of cell bodies with a modified silver cell-body staining (Merker 1983) that provides a high contrast between cell bodies and neuropil. The radiolabelled sections were then air dried and exposed against tritium-sensitive films (Hyperfilm, Amersham, Braunschweig, Germany) together with plastic tritium standards of known radioactivity concentrations (Microscales^®^, Amersham) for 4–18 weeks. The ensuing autoradiographs reveal the regional and laminar distribution of receptor binding sites.

### Image acquisition and analysis

Histological sections were scanned by means of a light microscope (Axioplan 2 imaging, ZEISS, Germany) equipped with a motor-operated stage controlled by the KS400^®^ and Axiovision (Zeiss, Germany) image analyzing systems applying a 6.3 × 1.25 objective (Planapo^®^, Zeiss, Germany), and a CCD camera (Axiocam MRm, ZEISS, Germany) producing frames of 524 × 524 µm in size, 512 × 512-pixel spatial resolution, with an in-plane resolution of 1 µm per pixel, and eight-bit gray resolution. These digitalized serial images were used for the qualitative cytoarchitectonic identification of distinct occipital areas in the macaque monkey brains.

Autoradiographs were digitized with an image analysis system consisting of a source of homogenous light and a CCD camera (Axiocam MRm, Zeiss, Germany) with an S-Orthoplanar 60-mm macro lens (Zeiss, Germany) corrected for geometric distortions, connected to the image acquisition and processing system Axiovision (Zeiss, Germany), to carry out densitometric analysis of binding site concentrations in the radioactive sections (Palomero-Gallagher and Zilles 2018; Zilles et al. 2002). Spatial resolution of the resulting images was 3000 × 4000 pixels (8-bit gray value resolution). Because these images only code gray values, which represent concentration levels of radioactivity, a scaling (i.e., a linearization of the digitized autoradiographs) was carried out to transform the gray values into fmol binding sites/mg protein using in house developed Matlab (The MathWorks, Inc. Natrick, MA) scripts. To provide a clear visualization of the regional and laminar receptor distribution patterns, digitized autoradiographs were linearly contrast enhanced and pseudo-color coded.

Receptor densities of each area were extracted from the linearized images by computing the surface below profiles defined vertically to the cortical surface using in house developed scripts for Matlab (The MathWorks, Inc. Natrick, MA) as previously described (Palomero-Gallagher et al. 2008; Palomero-Gallagher and Zilles 2018). Location of measuring sites, and assignment to a cytoarchitectonically identified area was ensured by comparison of the autoradiographs with the adjacent cell-body-stained sections.

### Identification of cortical areas

Selection of regions of interest for extraction of receptor densities in both macaque and human early visual areas was based on the architectonic identification of areas according to previously published criteria, and the analysis of multiple receptors in adjacent sections from the same brains. Specifically, in the macaque brain, areas V1–V3 and their subdivisions, as well as area V3A, dorsally and V4v ventrally were identified according to previously published cytoarchitectonic criteria and cortical maps (Felleman and Van Essen 1991; Niu et al. 2020; Van Essen et al. 1986). Areas marked as V3 and VP (Felleman and Van Essen 1991) correspond to our V3 subdivisions, mV3d and mV3v, respectively. A large portion of macaque V1 is also found on the lateral surface of the hemisphere, whereas human V1 is mainly located within the calcarine sulcus (Schira et al. 2012). Since retinotopic mapping has shown that the lower and upper visual fields are represented on the lateral surface in same manner as within the calcarine sulcus (Rosa 2002), we here concentrated on the sulcal portion of macaque V1.

In the human brain, areas V1 and V2 were identified according to Amunts et al. (2000), areas hOc3v and hOc4v on the ventral occipital cortex according to Rottschy et al. (2007), and areas hOc3d and hOc4d on the dorsal occipital cortex according to Kujovic et al. (2013). Areas hOc3d and hOc4d are the putative anatomical substrates of functionally defined areas V3d and V3A, respectively (Kujovic et al. 2013), and areas hOC3v and hOC4v are those of functionally defined areas V3v and V4v, respectively (Rottschy et al. 2007). Eickhoff et al. (2008) analyzed the receptor architecture of early visual areas in the human brain and besides confirming the cytoarchitectonically defined areas, identified dorso-ventral subdivisions within areas V1 and V2. Since visual inspection of the color coded receptor autoradiographs hinted at a comparable situation in the macaque brain, for each of these areas, densities were quantified in both species in a dorsally located region of interest (i.e., V1d, V2d) and in a ventrally located region of interest (i.e., V1v, V2v).

Note, that to avoid confusion, in the present analysis the prefix m- will be used to identify all monkey areas. Furthermore, to facilitate comparison between species, for human areas we will apply the functionally relevant nomenclature, albeit with the prefix h- (e.g., hV3A for area hOc4d).

### Statistical analysis of receptor densities

To determine if there were significant differences in receptor architecture between paired areas (dorsal and ventral subdivisions of the same visual region, or of adjacent areas from different hierarchical levels), stepwise linear mixed-effects models were performed separately for human and macaque visual areas. To ensure an equal weighting of each receptor in subsequent statistical analyses, receptor density values were normalized within each receptor type separately in human and macaque by applying the min–max scaling (Eq. 1).

1 $$z_{i} = \frac{{x_{i} - \min \left( x \right)}}{\max \left( x \right) - \min \left( x \right)} ,$$

where *x* is absolute receptor density*, i* represents an individual section, and *z* is the normalized data. Unless otherwise specified, normalization was performed separately in macaque and human data.

Statistical analyses were conducted using the R programming language (version: 3.6.3; Team 2013). For each species, the statistical testing involved three levels. In the first step, an omnibus test was carried out to determine whether there were differences across all areas when all receptor types are considered simultaneously (Eq. 2). The model consisted of fixed effects for area and receptor type, and human/macaque hemisphere was set as a random factor.

2 $$D_{a, r, b} = \alpha_{0} + \alpha_{1} A_{a} + \alpha_{2} R_{r} + \alpha_{3} A_{a} R_{r } + \beta_{1 } B_{b} \,,$$

where *D* is the receptor density, *A* is visual area, *R* is receptor type and *B* is human/macaque brain.

If the interaction effect between area and receptor type at the first step test was found to be significant, a second set of simple effect tests was performed for each receptor separately (i.e., 14 simple effect tests were performed in total) to determine whether there were significant differences across all areas for each receptor type. To correct for multiple comparisons in the second step tests, the false-discovery rate correction (Benjamini and Hochberg 1995) was performed (i.e., *p*-values were corrected for 14 comparisons).

Finally, for the receptor types that were found to show significant differences across all areas in the second step tests, a third set of post hoc tests were used to explore the paired areas that drove the statistical difference. For each receptor type, 28 post hoc tests were performed. To correct for multiple comparisons in the third step tests, we performed the false-discovery rate correction (Benjamini and Hochberg 1995) separately for each receptor type (i.e., *p*-values were corrected for 28 comparisons per receptor type).

### Multivariate cluster analyses

For each architectonically defined early visual area, we calculated mean areal densities (i.e., averaged over all cortical layers) of each of the 14 different receptors. To display the densities of multiple receptors within and between different cortical areas more intuitively, the ensuing densities were visualized for each area as a “receptor fingerprint”, i.e., as a polar coordinate plot simultaneously depicting the concentrations of all examined receptor types within that area (Palomero-Gallagher and Zilles 2018; Zilles et al. 2002).

Principal components (PCA) and hierarchical cluster analyses were carried out using in house developed Matlab (The MathWorks, Inc. Natrick, MA) scripts. Analyses were first carried out for macaque and human areas separately to identify the grouping of early visual areas in each species based on similarities in their receptor architecture. Receptor densities were normalized by *z*-scores for each receptor and species separately to ensure an equal weighting of receptors expressed at overall high and low densities. The Euclidean distance was used in the hierarchical cluster analysis as a measure of (dis)similarity of areas since it best captures the differences in size and shape between fingerprints, and the Ward linkage algorithm was chosen as the linkage method, since in combination with the Euclidean distance it resulted in the maximum cophenetic correlation coefficient as compared to any combination of alternative linkage methods and measurements of (dis)similarity (Palomero-Gallagher et al. 2009). The optimal number of clusters, *k*, for the K-means algorithm was determined by clustering the data with *k* from 1 to 9. For each clustering of the data, the squared Euclidean distance between the data points and their respective centroids, i.e., distortion, was calculated and plotted against each *k* (Rousseeuw 1987). We also sought to determine similarities between receptor types by how their expression levels varied across areas. To this purpose, we transposed the matrices used for the clustering of areas based on differences in their receptor fingerprints, so that receptor densities were normalized by *z*-scoring for each area and species separately, and carried out a second set of PCA, hierarchical cluster and K-means analyses separately for the macaque and human brains.

Finally, to address the question of homologies between human and macaque visual areas, a species-combined PCA was conducted as previously described (Sherwood et al. 2004). Note that prior to this PCA, a species-combined normalization was performed. That is, within each receptor, the density for all macaque and all human areas were jointly normalized by *z*-scores.

## Results

**Fig. 1:**
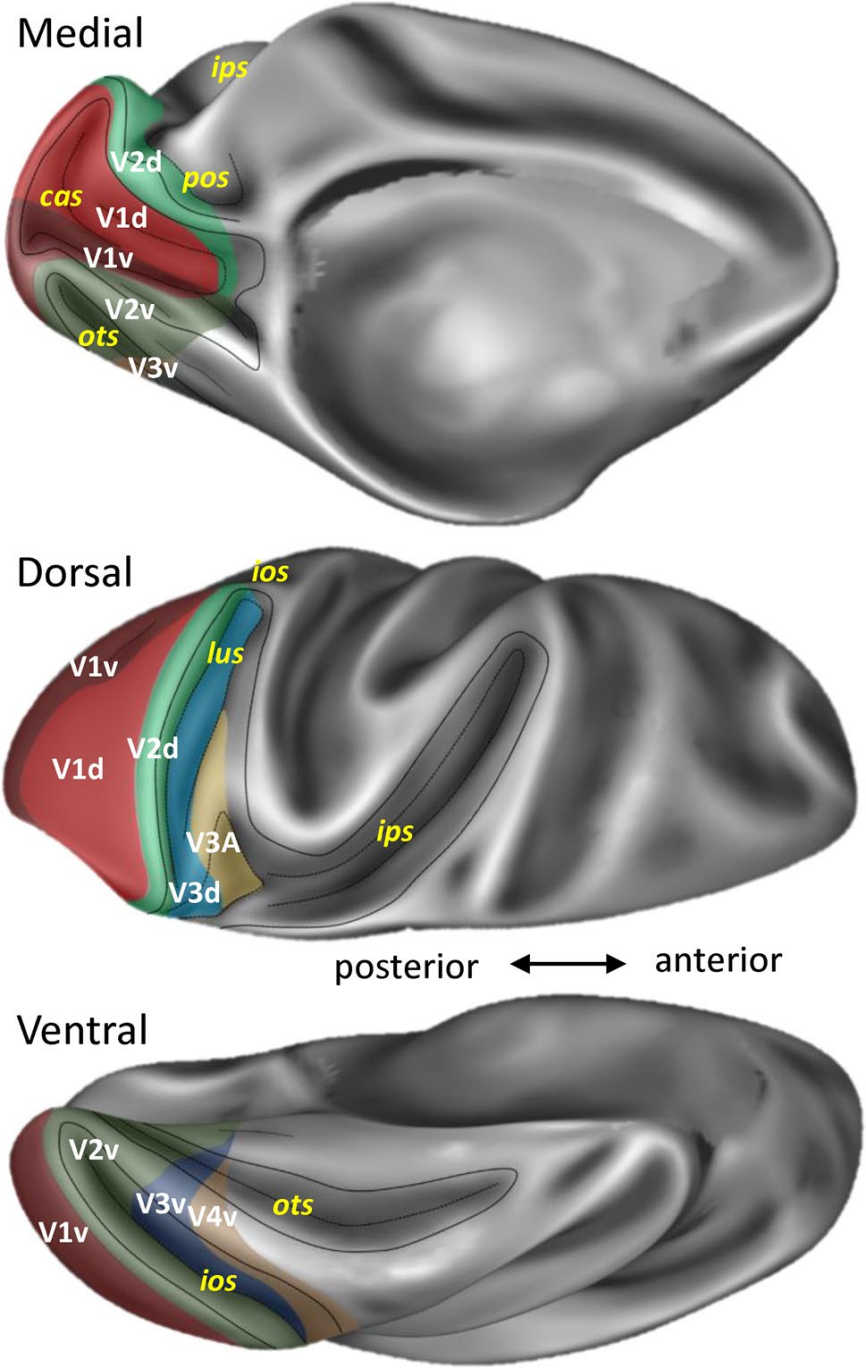
Topography of the eight cyto- and receptor architectonically distinct areas identified in the macaque brain depicted on the Yerkes 19 surface. (map made publicly available at https://balsa.wustl.edu/study/l77k6). Abbreviations: *cas* calcarine sulcus, *ips* intraparietal sulcus, *pos* parieto-occipital sulcus, *ots* occipito-temporal sulcus, *lus* lunate sulcus, *ios* inferior occipital sulcus

Eight subdivisions of the early visual cortex were identified and receptor architectonically characterized in the macaque monkey (mV1d, mV2d, mV3d, mV3A, mV1v, mV2v, mV3v, and V4v; Fig. 1) and the human (hV1d, hV2d, hV3d, hV3A, hV1v, hV2v, hV3v, and hV4v) brain.

**Fig. 2:**
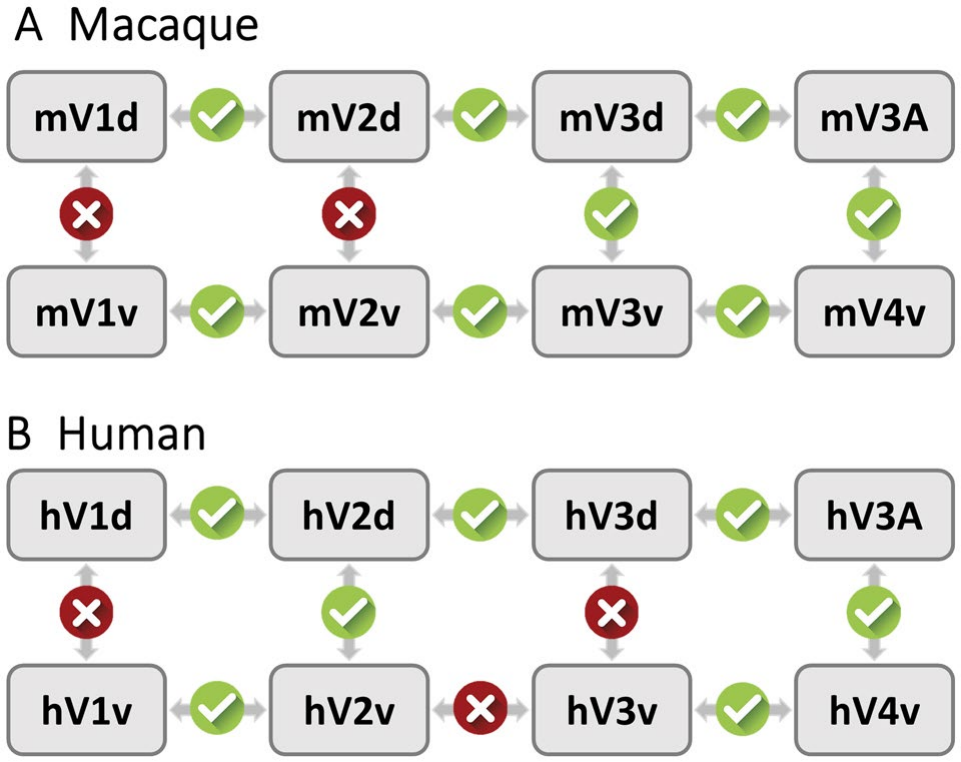
Schematic representation of the strategy used for statistical testing of differences in mean receptor densities between early visual areas. For each species, the statistical testing process involved three steps. (1) an omnibus test was carried out to determine whether there were differences across all areas when all receptor types are considered simultaneously. (2) The simple effect tests were performed for each receptor separately to determine if this receptor type contributed to the distinction of early visual areas. (3) For those receptor types that were found to show significant differences across all areas in the second step tests, post hoc tests were used to explore which paired areas drive the statistical difference. Arrows indicate pairs of areas compared. Results of the statistical analysis (after correction of *p*-values) are indicated by checkmarks in green circles (significant finding) or crosses in red circles (the two areas do not differ significantly in their receptor architecture). For information concerning which receptor types contributed to the significance, see Supplementary Fig. 5. Statistical values for the first level test are provided in Supplementary Table 2, those of the simple effects in Supplementary Table 2, and those of post hoc tests in Supplementary Tables 4 and 5 (macaque and human data, respectively)

Stepwise linear mixed-effects models were performed for macaque and human brains to determine whether there were significant differences in receptor densities between adjacent pairs of areas along the visual hierarchy separately for the dorsal and ventral streams (e.g., from V1d through V2d to V3A), as well as between the dorsal and ventral components of a specific hierarchical level (e.g., V1d vs V1v), and if so, which receptor types contributed to these distinctions. The interaction effect between area and receptor type was found to be significant in the first level test (Supplementary Table 2), and second level tests for each receptor type separately revealed that density differences in all receptors except for M_1_ and D_1_ in macaques and GABA_B_ and D_1_ in humans contribute to the segregation of early visual areas (Supplementary Table 3). The results of the third level tests, which served to identify which paired areas drove the statistical difference and will be described in the following paragraphs, and are listed in Supplementary Tables 4 and 5 (macaque and human brains, respectively) and graphically displayed in Fig. 2 and Supplementary Figs. 4–5.

### Cytoarchitecture and receptor distribution patterns

#### Subdivisions of area V1

**Fig. 3:**
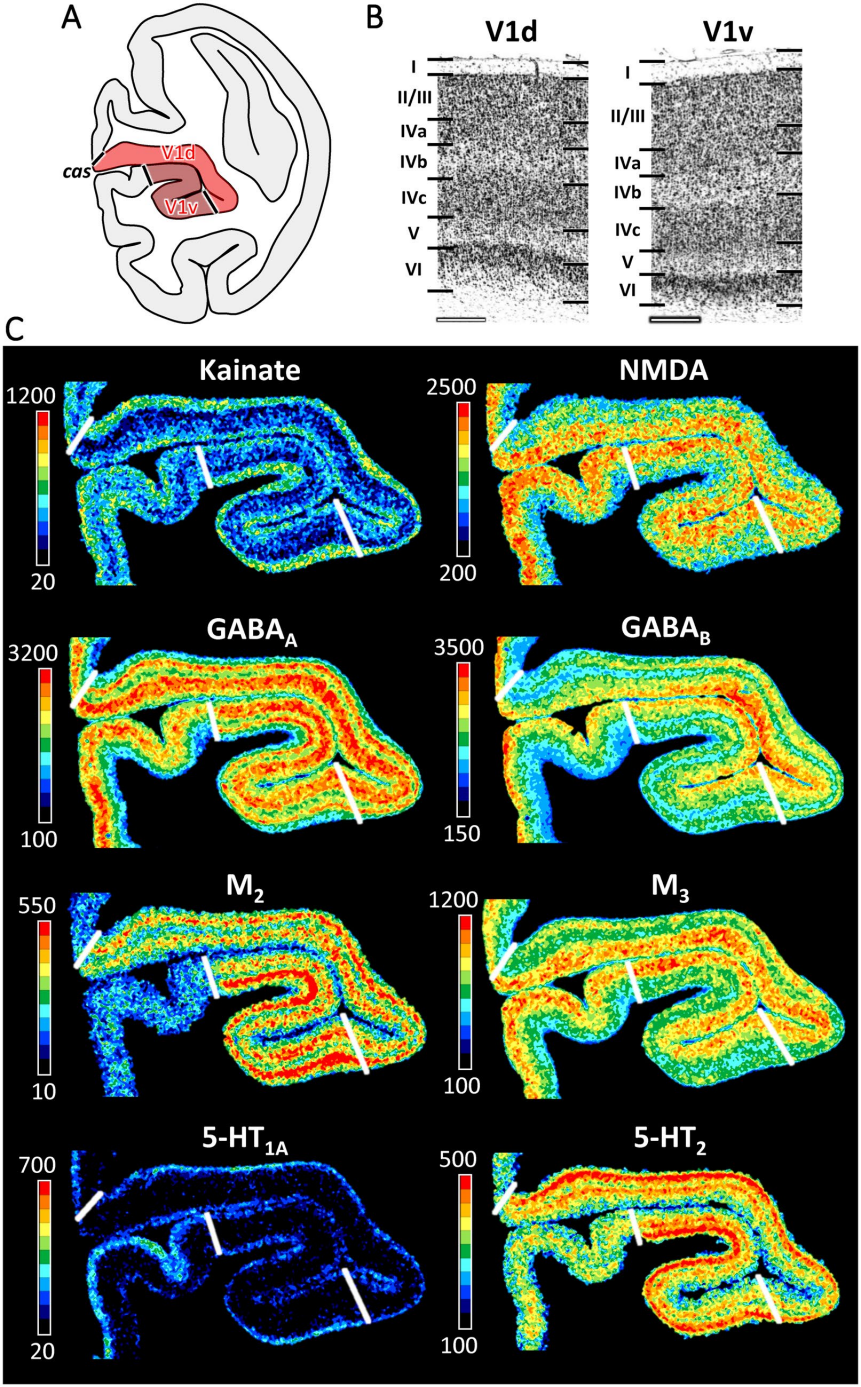
Cyto- and receptor architecture of macaque primary visual area V1. **A**: Schematic drawing of a coronal section through the macaque brain showing the position of dorsal (mV1d) and ventral (mV1v) subdivisions of V1 within the calcarine sulcus. **B**: High-resolution photomicrographs of cytoarchitectonic features of areas mV1d and mV1v. Scale bar 300 µm. Roman numerals indicate cytoarchitectonic layers. **C**: Exemplary sections depicting the distribution of kainate, NMDA, GABA_A_, GABA_B_, M_2_, M_3_, 5-HT_1A_ and 5-HT_2_ receptors. The color bar next to each autoradiograph codes for receptor density in fmol/mg protein and borders are indicated by the white lines. Distribution patterns of the remaining receptors are shown in Supplementary Fig. 1. For abbreviations see Fig. 1

Dorso-ventral heterogeneities were recognized in area V1 within the calcarine sulcus (*cas*) based on cytoarchitectonic differences in sublayers IVa and IVb (Fig. 3; Supplementary Fig. 1). The dorsal subdivision (mV1d), has a more prominent layer IVa when compared to its ventral counterpart (mV1v). Furthermore, mV1d has more densely packed, and larger pyramids in layer IVb than does mV1v. Differences between mV1d and mV1v were confirmed by the receptor architectonic analysis (Fig. 3; Supplementary Fig. 1). Lower GABA_A_, GABA_B_, GABA_A_/BZ and M_3_ receptor densities were found in the infragranular layers of mV1v than in those of mV1d, whereas the opposite holds true for the kainate, 5-HT_1A_, and 5-HT_2_ receptors. However, these differences did not reach the level of significance at the mean areal (i.e., densities averaged over all cortical layers) level.

In addition to differences between the dorsal and ventral banks of macaque V1, a modular distribution throughout macaque V1 was particularly obvious for the M_2_ receptor, and to a lesser extent for the GABA_A_ and 5-HT_2_ receptors (Fig. 3; Supplementary Fig. 1). Furthermore, GABA_A_/BZ, GABA_B_, M_1_, and M_2_ densities are higher in the lateral than in the medial portion of mV1d, and the opposite holds true for M_3_ receptors in mV1v.

#### V1/V2 border and subdivisions of area V2

**Fig. 4:**
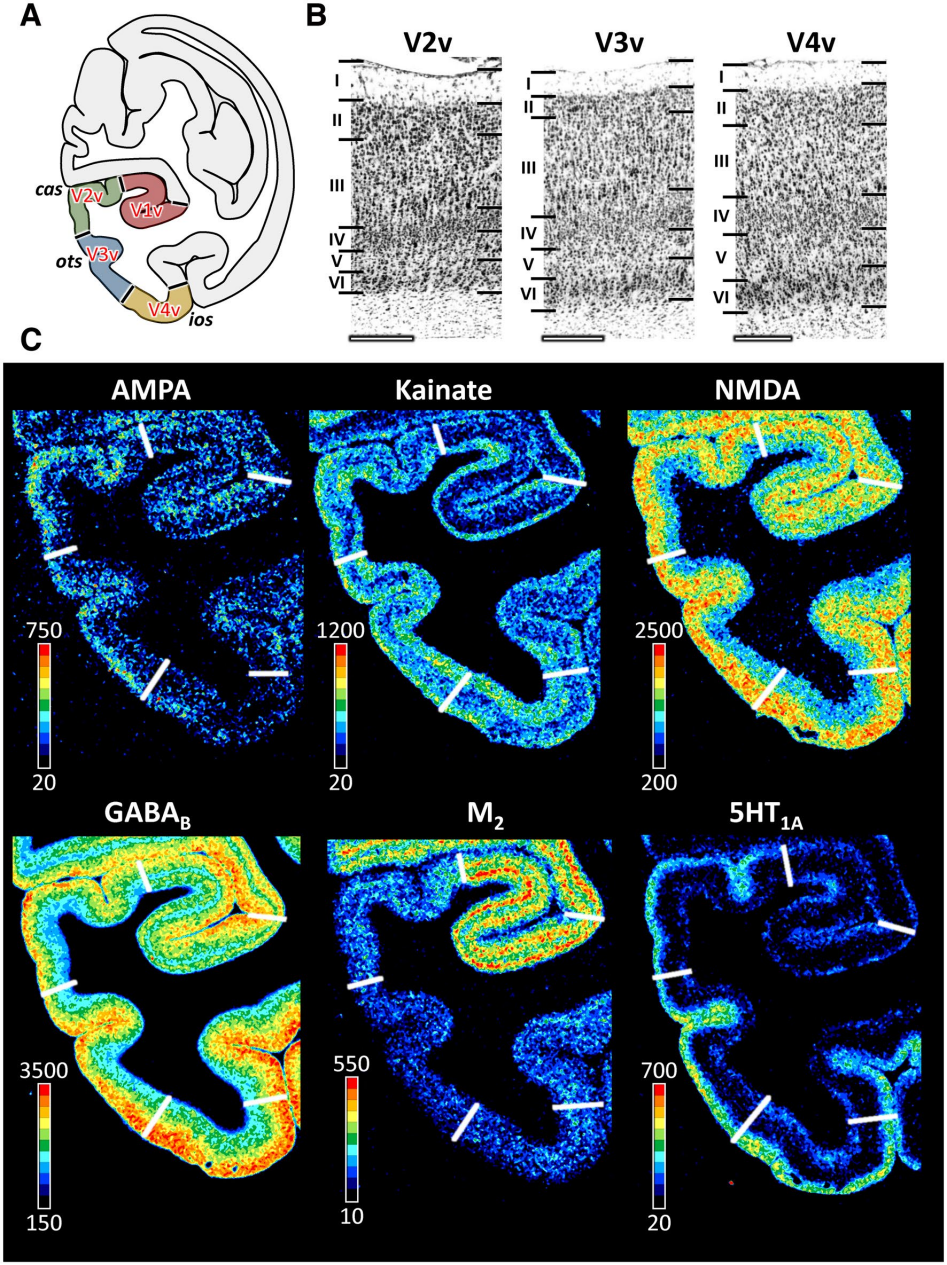
Cyto- and receptor architecture of macaque ventral early extrastriate visual areas. **A**: Schematic drawing of a coronal section through the macaque brain showing the position of areas mV2v, mV3v and mV4v. **B**: High-resolution photomicrographs of cytoarchitectonic features of areas mV2v, mV3v and mV4v. Scale bar 300 µm. Roman numerals indicate cytoarchitectonic layers. **C**: Exemplary sections depicting the distribution of AMPA, kainate, NMDA, GABA_B_, M_2_ and 5-HT_1A_ receptors. The color bar next to each autoradiograph codes for receptor density in fmol/mg protein and borders are indicated by the white lines. Distribution patterns of the remaining receptors are shown in Supplementary Fig. 2. For abbreviations see Fig. 1

**Fig. 5:**
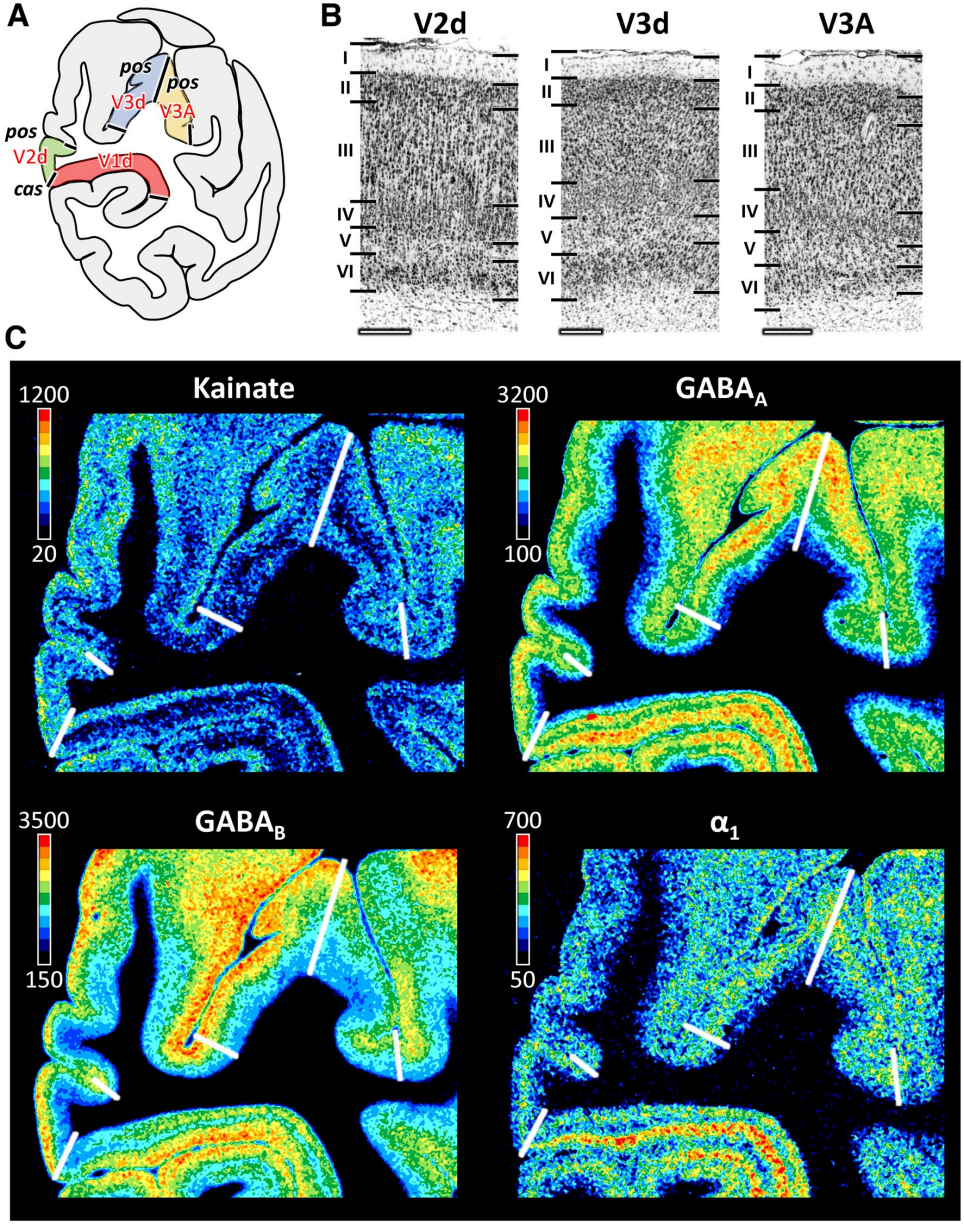
Cyto- and receptor architecture of macaque dorsal early extrastriate visual areas. **A**: Schematic drawing of a coronal section through the macaque brain showing the position of areas mV2d, mV3d and mV3A. **B**: High-resolution photomicrographs of cytoarchitectonic features of areas mV2d, mV3d and mV3A. Scale bar 300 µm. Roman numerals indicate cytoarchitectonic layers. **C**: Exemplary sections depicting the distribution of kainate, GABA_A_, GABA_B_ and α_1_ receptors. The color bar next to each autoradiograph codes for receptor density in fmol/mg protein and borders are indicated by the white lines. Distribution patterns of the remaining receptors are shown in Supplementary Fig. 3. For abbreviations see Fig. 1

Area mV2 is located anterior to mV1 as a continuous cortical belt (Fig. 1). The boundary between both areas is the clearest cytoarchitectonic border due to the sharp change from a tripartite layer IV in V1 to a homogeneous granular layer in V2 (Figs. 3, 4, 5; Supplementary Figs. 1–3). The border between layers II and III, as well as that between layers IV and V, is sharper in the portion of mV2 located dorsal to V1 (i.e., in mV2d) than in mV2v (Figs. 3 and 4). Significant differences in receptor architecture between V1 and V2 were found for most receptor types (Supplementary Fig. 4). Area mV2d contains lower NMDA, M_2_, M_3_, α_2_ and 5-HT_2_, but higher kainate and 5-HT_1A_ receptor densities than does mV1d. Ventrally, mV2v contains lower NMDA, GABA_A_, M_2_, and α_2_ but higher 5-HT_1A_ receptor densities than does mV1v.

Although differences between mV2d and mV2v were evident at the laminar level, with infragranular layers of the former area presenting higher kainate, NMDA, GABA_B_, and M_1_ receptor concentrations than those of the latter area (Figs. 4 and 5, Supplementary Figs. 2 and 3), no significant differences were found at the mean areal level (Supplementary Fig. 4).

In the human brain, we found a comparable pattern of significantly different receptor densities between adjacent subdivisions of areas V1 and V2 to that described for the macaque (Supplementary Fig. 4). NMDA, M_2_, M_3_, α _2_ densities were also higher, and 5-HT_1A_ densities lower in hV1 than in hV2. However, kainate densities did not differ significantly between both areas in the human brain, but GABA_A_ densities were significantly higher in hV1 than in hV2. Furthermore, when comparing the dorsal and ventral components of V2, we found a significantly higher 5-HT_2_ receptor density in hV2d than in hV2v.

#### V2/V3 border and subdivisions of area V3

Dorsal to mV2d, mV3d was identified within the posterior region of the sulcal complex of the *pos*, and was followed laterally by mV3A, which lies in the fundus of the *ips*/*pos* junction (Fig. 1). Due to inter-individual variability in the extent of area mV3d, the mV3d/mV3A border can be found either on the rostral wall of the anectant gyrus, on the apex of the gyrus, or on its posterior wall. On the ventral occipital surface, mV3v replaces mV2v between *ios* and *ots*, and is also found within these sulci. Area mV4v is located ventral and anterior to mV3v, on the rostral wall of the *ios*, and extending onto the ventrolateral surface of the hemisphere. Whereas mV3d and mV3v from a continuous cortical belt around mV2, areas V3A and V4v do not share a common border (Fig. 1).

Areas mV3d and mV3v can be clearly delineated from mV2d and mV2v, respectively, due to the more prominent lamination, particularly concerning cell density in layer IV and the columnar differentiation in layer III, in the latter than in the former areas (Figs. 4 and 5). The clear bilaminar distribution of M_2_ receptors in mV2v is no longer visible in mV3v (Fig. 4), and mean kainate receptor densities were significantly higher in mV3v than in mV2v (Figs. 4 and 5, Supplementary Fig. 4). Dorsally, mV2d contains a significantly higher kainate receptor density than does mV3d (Figs. 4 and 5, Supplementary Fig. 4).

Compared to mV3v, mV3d has a more prominent layer II and, in general, a clearer lamination (Figs. 4 and 5). These two areas also differ significantly in their kainate and 5-HT_1A_ receptor densities, which are higher in mV3v than in mV3d (Figs. 4 and 5, Supplementary Figs. 2–4).

The main cytoarchitectonic difference between mV3A and mV3d is the clear sublamination of layer V in mV3A, but not in mV3d. Furthermore, a slight increase in the size of layer IIIc pyramids is noticed when moving from mV3d to mV3A (Fig. 5). For most receptor types differences between mV3d and mV3A were most prominent in the supragranular layers (Fig. 5, Supplementary Fig. 3). mV3d contains a lower 5-HT_1A_ receptor density than does mV3A (Supplementary Fig. 4).

Cytoarchitectonic analysis revealed that area mV4v has wider and more densely packed layers II and IV compared to area mV3v (Fig. 4). Furthermore, as shown in Fig. 3 and Supplementary Fig. 2, area V4v has lower AMPA and GABA_A_, but higher GABA_A_/BZ, GABA_B_, and M_1_ densities than V3v. However, only the difference in the density of AMPA receptors reached the level of significance (Supplementary Fig. 4).

In the human brain significant differences were also found between all pairs of adjacent areas belonging to the dorsal stream (Supplementary Fig. 4). As in the macaque brain, densities in hV3d were significantly lower than those in hV2d or hV3A. Within the ventral stream, and in contrast with findings in the macaque brain, no significant differences were found between hV2v and hV3v. However, as described for the macaque brain, the significantly higher densities were found in hV3v as compared to hV4v.

### Multivariate analyses of receptor fingerprints

**Fig. 6:**
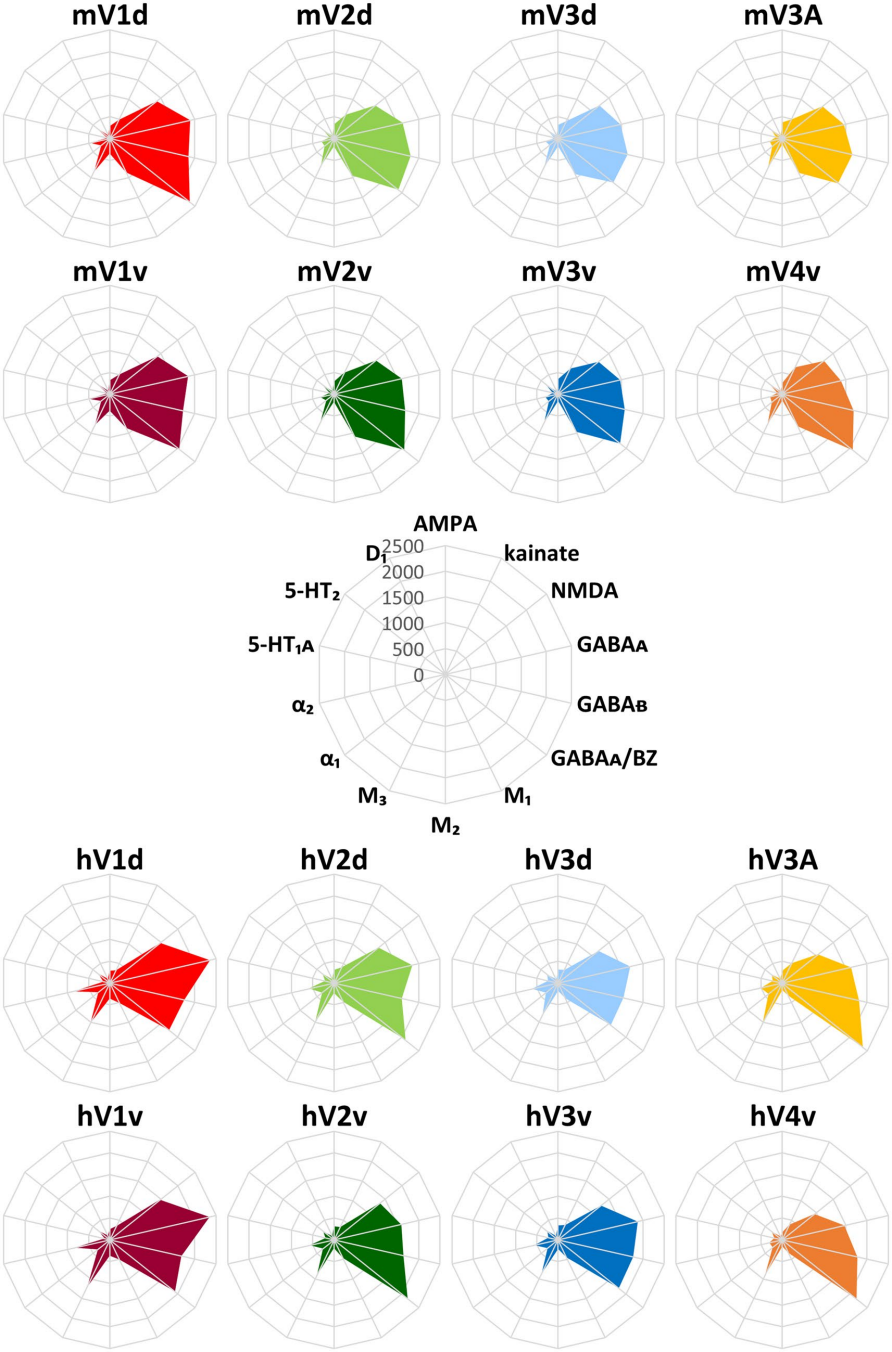
Receptor fingerprints of the early visual areas in the macaque monkey and human brain. Absolute receptor densities are given in fmol/mg protein. The positions of the different receptor types and the axis scaling are identical in all areas, and specified in the polar plot in the middle of the figure. Data are publicly available via the EBRAINS platform of the Human Brain Project (https://search.kg.ebrains.eu/instances/Project/e39a0407-a98a-480e-9c63-4a2225ddfbe4) and under https://balsa.wustl.edu/study/l77k6

Figure 6 shows the receptor fingerprints of areas analyzed in the present study in the early visual cortex of both macaque monkey and human brains, and Supplementary Table 6 provides numeric values. The corresponding normalized data are presented in Supplementary Fig. 6 and Supplementary Table 7. The absolute mean receptor concentration varies considerably between the different receptor types in each area. In both species, GABA_A_ and GABA_B_ receptors, as well as GABA_A_/BZ binding sites are present at the highest absolute densities, whereas lowest absolute densities are reached by the 5-HT_1A_ and D_1_ receptors.

**Fig. 7:**
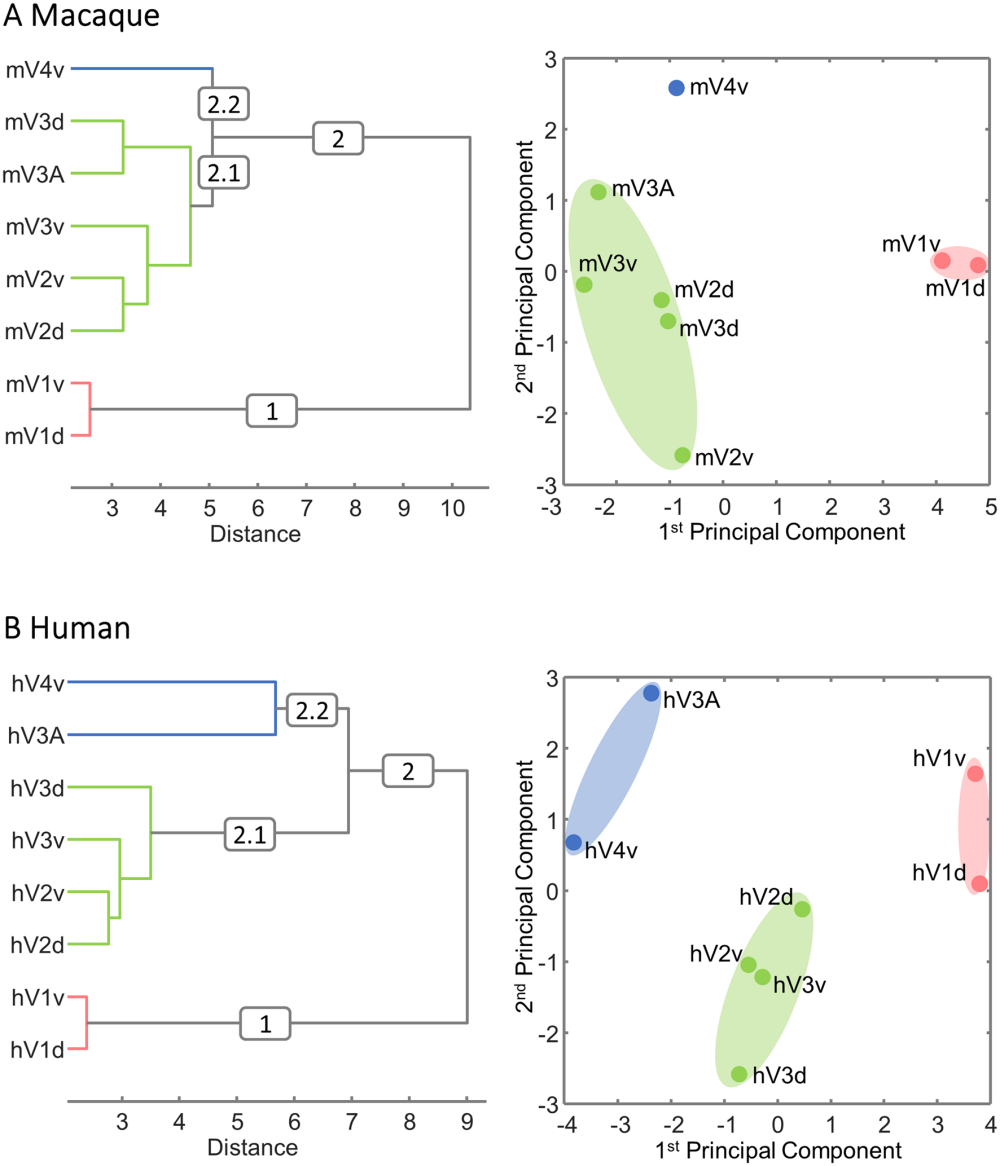
Hierarchical clustering and principal component analysis (PCA) aiming to determine clustering of visual areas based on (dis)similarities in their normalized receptor fingerprints. *k*-means clustering and elbow analysis showed three as the optimal number of clusters for both species. **A**: Macaque monkey visual areas **B**: Human visual areas. Receptor densities were normalized separately for macaque and human visual areas, and data are provided in Supplementary Table 7

Two sets of hierarchical clustering and principal component analyses were performed with each the macaque and the human data. The first set of analyses aimed to visualize the degree of (dis)similarity in the normalized fingerprints of early visual areas (Fig. 7). The *k*-means analysis and elbow plots clearly indicated that *k* = 3 provided the optimal trade-off between number of clusters and distortion for both the macaque and the human brain (Supplementary Fig. 7). The second set of analyses aimed to identify (dis)similarities between receptors in their expression levels across visual areas (Supplementary Fig. 8), and *k* = 2 was found to be the optimal number of clusters for the macaque brain, whereas that for the human brain was *k* = 4 (Supplementary Fig. 9).

In the macaque monkey (Fig. 7A), cluster 1 contained the two subdivisions of mV1, which separated very early from the remaining areas (cluster 2). Within cluster 2, areas mV2d, mV2v, mV3d, mV3v and mV3A are found in one group (cluster 2.1), whereas area mV4v is separated from the other early visual areas to form an isolated cluster 2.2. The segregation of clusters 1 and 2 was also confirmed by the 1st principal component of the PCA, and that of clusters 2.1 and 2.2 by the 2nd principal component (Fig. 7A). The analyses aiming to reveal which receptors can be grouped based on how their densities change across the examined areas (Supplementary Fig. 8A) indicated a segregation of AMPA, kainate, M_1_, α_1_ and 5-HT_1A_ receptors (cluster 2) from the remaining examined receptor types (cluster 1), which was confirmed by differences along the 1st principal component of the PCA (Supplementary Fig. 8A).

In the human brain, areas hV1d and hV1v also grouped together as a single cluster (cluster 1, Fig. 7B). Areas hV2d, hV2v, hV3d and hV3v are all found in a single cluster (cluster 2.1), and areas hV3A and hV4v are grouped in cluster 2.2. The 1st principal component of the PCA clearly segregated these three clusters, whereas the 2nd principal component more strongly reflected differences between hV2d, hV2v, hV3d and hV3v and the remaining areas (Fig. 7B). Clustering of the receptors according to variations in their distribution patterns across visual areas (Supplementary Fig. 8B) revealed four clusters: Cluster 1.1 contained the NMDA, GABA_A_, M_1_, M_2_, α_2_ and 5-HT_2_ receptors; cluster 1.2 the M_3_, α_1_ and D_1_ receptors; cluster 2.1 the AMPA and 5-HT_1A_ receptors; cluster 2.2 the kainate and GABA_B_ receptors as well as the GABA_A_/BZ binding sites. In the PCA, AMPA and 5-HT_1A_ were separated from the remaining receptors by differences along the 2nd principal component, whereas clusters 1.1, 1.2 and 2.2 were segregated by the 1st principal component.

**Fig. 8:**
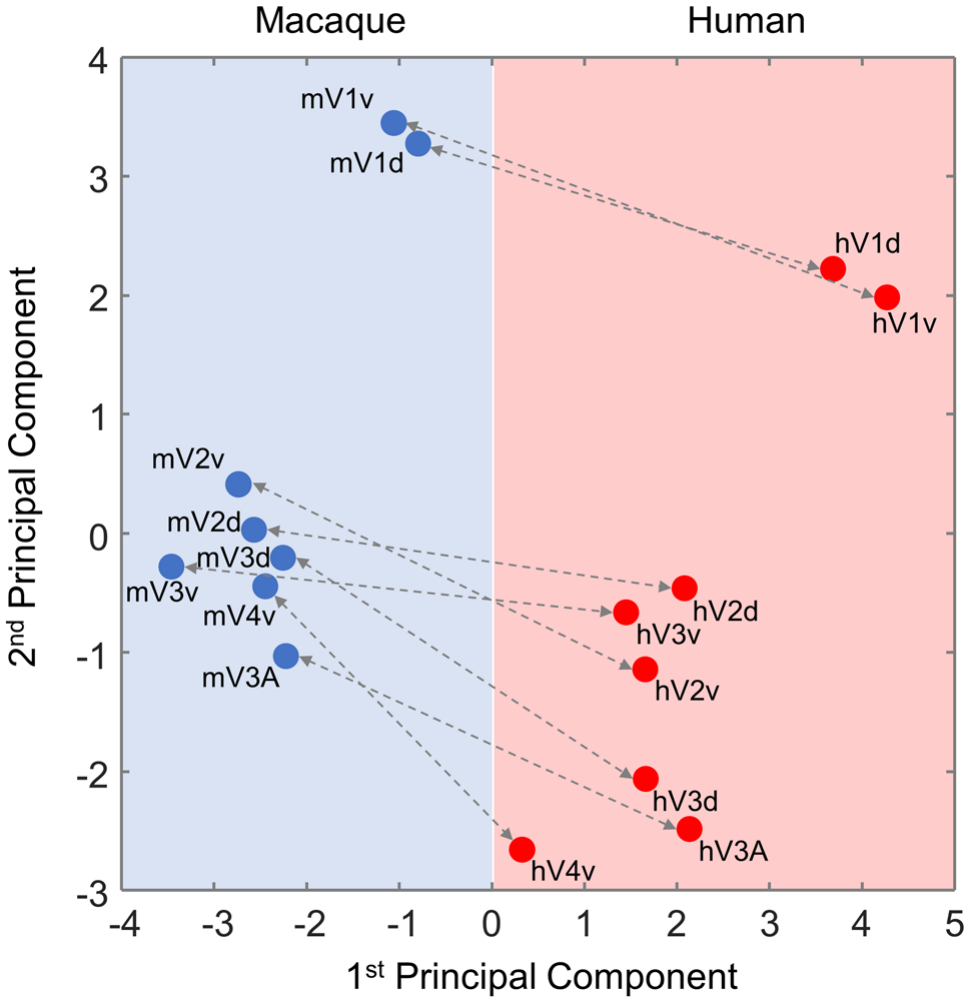
A species-combined principal component analysis (PCA) of the receptor densities in human (red) and macaque (blue) primary and early extrastriate visual areas. Receptor densities were normalized after combining both species into the same space, and data are provided in Supplementary Table 8

Finally, to address the issue of comparability between homolog areas in each species, a species-combined PCA was performed (Fig. 8). The 1st principal component clearly segregates human and macaque areas, whereas the 2nd principal component generally reflects differences in the fingerprints associated with the hierarchical processing level of each area.

## Discussion

We here present the first quantitative analysis of the distribution and inter-individual variability in the densities of 14 neurotransmitter receptors in the cytoarchitectonically identified macaque primary visual area V1, early visual areas V2d, V2v and V3v, as well as of higher visual area V4v, and compare our results with data obtained from the human brain (Eickhoff et al. 2007, 2008; Zilles and Palomero-Gallagher 2017b). Multivariate analyses of the receptor densities extracted from the identified areas revealed that although the receptor fingerprints of monkey early occipital areas differ from those of their counterparts in the human brain, within each species the area-specific differences in receptor densities reflected the hierarchical processing level of each area in a comparable manner.

We analyzed receptors for classical neurotransmitters because, unlike neuropeptides, classical neurotransmitters are actively involved in conveying information across a synapse, and unlike non-classical neurotransmitters, they mediate unidirectional anterograde signal transmission. With the receptors analyzed here, we cover a representative sample of the ionotropic/metabotropic and excitatory/inhibitory receptor types to which the major classical neurotransmitters glutamate, GABA, acetylcholine, noradrenaline and serotonin can bind, and which serve to explain the diversity of signal amplification and processing levels as well as time scales at which neurochemical signalling takes place in the mammalian brain (Palomero-Gallagher and Zilles 2018). Furthermore, these receptors have been shown to be evolutionarily conserved in the primary sensory areas of human and macaque monkey brains (Zilles and Palomero-Gallagher 2017a).

### Receptor architectonic subdivisions of cytoarchitectonically identified visual areas in the macaque brain

Area V1 is the cytoarchitectonically most differentiated isocortical area in the primate brain, with a unique sublamination of layer IV (Zilles et al. 2015b). This cytoarchitectonic uniqueness is mirrored by its receptor architecture, which clearly reveals the border to V2, as revealed not only in the macaque (present results; Hendry et al. 1990; Rakic et al. 1988; Rakic and Lidow 1995; Rosier et al. 1991; Zilles and Clarke 1997; Zilles and Palomero-Gallagher 2017a) but also in the vervet brain (Takemura et al. 2020).

Layer-specific differences in receptor densities enabled the definition of qualitative dorsal and ventral components of mV1 within the calcarine sulcus, as well as medio-lateral density gradients within each of these compartments. This heterogeneous receptor distribution probably represents the molecular underpinning of the fact that visual information from the upper and lower, as well as from the peripheral and central, visual fields is known to processed separately in primate V1 (Dougherty et al. 2003; Gattass et al. 2005; Previc 1990; Silson et al. 2018; Van Essen et al. 1986). Furthermore, since mV1d sends topographically organized projections to mV2d and mV3d, whereas mV1v projects to mV2v, but not to mV3v (Van Essen et al. 1986), this retinotopic organization is propagated through early extrastriate visual areas, and also reaches areas of the posterior inferotemporal and dorsal occipitotemporal cortex (Kolster et al. 2014; Zhu and Vanduffel 2019).

The modular distribution of M_2_, GABA_A_, and 5-HT_2_ receptors within macaque V1 resembles the previously described blobs and interblobs revealed by cytochrome oxidase staining (Horton and Hubel 1981; Wong-Riley 1979), as well as the periodical distribution of GABA_A_ receptors in the human brain (Zilles and Schleicher 1993). Although the functional meaning of blobs and interblobs has been controversially discussed in the literature, they are commonly thought to be associated with differential color domains and orientation-selective processes (Lu and Roe 2008).

Similar to V1, area V2 in the monkey has been described as a cytoarchitectonically homogeneous region (de Sousa et al. 2010), and we detected no significant differences in receptor densities at the mean areal level. However, we found a trend towards higher kainate, NMDA, GABA_B_, and M_1_ densities in the infragranular layers of mV2d, as well as higher 5-HT_1A_ but lower M_2_ concentrations in its supragranular layers than in the corresponding layers of mV2v. These qualitative differences would be in accordance with the dorso-ventral asymmetry in connectivity patterns of V2. Whereas V2d and V2v project back to V1 and forward to dorsal and ventral parts of V3 (Gattas et al. 1997), output to area V4t was found to originate in the dorsal part of V2, but not in V2v (Gattass et al. 1997, 2005). Furthermore, V2 encompasses dorsal and ventral functional subdivisions in which the inferior and superior contralateral quadrants are represented, respectively (Gattass et al. 1981), and these subdivisions also differ in the length and orientation of their cytochrome oxidase positive stripes (Olavarria and Van Essen 1997).

Cortex located immediately rostral to V2 has been designated as the “third visual complex”, and encompasses our areas V3v, V3d, and V3A (Rosa et al. 2005; Zeki 1978), where area V3v has also been designated as area VP (de Sousa et al. 2010; Hof and Morrison 1995; Zilles and Clarke 1997). The receptor architecture of areas V3d and V3A, which are located at the junction of the intraparietal and parieto-occipital sulci, was comprehensively characterized in a recent mapping study of the macaque intraparietal sulcus, and the same sample was used as for the present analysis (Niu et al. 2020). Our data confirm and expand on a study by Kötter et al. (2001) on the relationship between area-specific differences in receptor densities and connectivity patterns in multiple areas of the macaque monkey brain, including visual areas analyzed here, since their analysis of the visual cortex only included the AMPA, kainate, GABA_A_, M_1_, M_2_ and 5-HT_2_ receptors, and they only extracted densities from a single macaque hemisphere (which was not included in the present analysis).

### Similarities and differences in the receptor architecture of macaque and human early visual areas

We found the fingerprints of macaque visual areas to differ in shape from those of their human homologs, indicating species-specific differences in the balance between the analyzed receptor types of the GABAergic system. E.g., whereas GABA_A_/BZ binding site densities were higher than GABA_A_ receptor densities in mV1d and mV1v, the opposite holds true for hV1d and hV1v. Human and macaque V1 are also known to differ in their laminar distribution pattern of cytochrome oxidase activity in layers IVa and IVb, and in the organization of input from the magno- and parvocellular projections from LGN (Preuss et al. 1999) which has been interpreted as suggesting an evolutionary shift in the organization of LGN input to the primary visual cortex and reflecting different mechanisms of motion processing in humans than in non-human primates (Orban et al. 2004).

In both species, primary visual area V1 significantly differed from V2 by a higher mean density of M_2_ and α_2_ receptors, but a lower one 5-HT_1A_ receptors. These differences are in accordance with previous receptor architectonic reports in the human visual cortex (Eickhoff et al. 2007, 2008; Zilles and Palomero-Gallagher 2017b), and are also supported by qualitative descriptions in the macaque brain (Rakic et al. 1988; Rakic and Lidow 1995). Notably, the hierarchical cluster analysis carried out to identify groupings of receptors based on (dis)similarities in their expression levels throughout visual areas revealed for both macaque and human brains that the 5-HT_1A_ receptors were located cluster 2, whereas the M_2_ and α_2_ receptors were in cluster 1. Additionally, in macaques V1 presented significantly higher 5-HT_2_ levels than V2, whereas human V1 and V2 differed in GABA_A_ receptor densities, thus highlighting possible interspecies differences in the molecular mechanisms subserving information transfer between V1 and early visual areas.

Given the differences between V1 and V2, it is not surprising that the hierarchical cluster analysis and the 1st principal component of the PCA clearly segregated the fingerprints of human and macaque primary subdivisions from the rest of the visual areas (Fig. 7). Furthermore, as shown by the combined PCA, both species have in common that segregation along the 2nd principal component reflected differences in fingerprints which are associated with the hierarchical processing level of each area. Thus, the transition that the molecular structure of early visual areas undergoes when moving from the primary visual cortex through V2 and V3, and up to V3A and V4v, is comparable in the macaque and human brains.

There were species differences, however, concerning the segregation pattern of V3A, and they were also confirmed by the hierarchical clustering analysis: in the macaque brain, mV3A clustered with mV3d, but in the human brain it was clearly separated, together with hV4v, from lower level visual areas (Fig. 7). Receptor fingerprints of hV3A and hV4v differ in shape from the rest of areas in the balance between GABA_A_/BZ and GABA_B_ receptors, indicating functionally specific areas, which represent different hierarchical levels within the visual system. Interestingly, differences in kainate receptors were found to be significant in the monkey brain, but not human; i.e., mV3A and mV3d expressed significantly lower kainate densities than the surrounding areas. Pre- and postsynaptic kainate receptors are important for neurotransmission regulation, and seem to be involved in short- and long-term plastic phenomenon, highlighting their crucial role in synaptic signaling (Lerma 2003).

Area V3A represents an intermediate region in visual processing between lower level areas V1–V3 and higher visual areas of the dorsal and ventral streams (Tootell et al. 1997), since it shares connections with areas in the parietal and the temporal cortex (Felleman and Van Essen 1991). Interestingly, functional studies in humans associated area V3A with motion processing (Tootell et al. 1997), while similar studies in monkeys described area V3d as being more sensitive to motion than area V3A (Tootell et al. 1997; Tolias et al. 2001; Vanduffel et al. 2002), suggesting that area V3A plays different roles in humans and monkeys (Orban et al. 2003, 2004; Tootell et a﻿l. 1997), and the differing clustering patterns of area V3A in the human and macaque visual systems described in the present study provide further support for this hypothesis. However, monkey V3A has a similar retinotopic organization to that of human V3A, with a complete representation of the visual field separated by the horizontal meridian (Brewer et al. 2002; Fize et al. 2003; Tootell et al. 1997; Gattass et al. 1988), and in both species is associated with the processing of stereoscopic stimuli (Backus et al. 2001; Tsao et al. 2003). The fact that our clustering analyses did not result in a clear segregation of areas V2d, V2v, V3d and V3v could indicate that crosstalk between areas of the dorsal and ventral streams not only occurs at hierarchically higher processing levels (Van Polanen and Davare 2015), but that there is already a strong interconnectivity between both streams at very early stages of the processing of visual stimuli.

In the macaque, area mV4v formed its own cluster, not only due to differences in the shape of fingerprints, but also to the fact that its fingerprint is the smallest of all analyzed areas. However, in humans hV4v was found to cluster with hV3A, indicating that the receptor fingerprint of mV4v differs more from those of the remaining macaque extrastriate visual areas than does hV4v from the remaining human extrastriate visual areas. This latter fact seems to be driven by species-specific differences since the overall receptor balance in hV4v is driven by the high densities of the GABAergic receptors. Primate area V4v constitutes a mid-level visual processing region that receives input primarily from area V2 and sends output to the inferior temporal cortex (Tootell et al. 1997) as well as topographically organized feedback projections to V2 and V3 (Ungerleider et al. 2008). It has been characterized as a color-sensitive area representing the dorsal half of the visual field (Felleman and Van Essen 1991; Gattass et al. 1988; Zeki 1978). A functionally comparable region was defined in the human brain based on in vivo retinotopic imaging (DeYoe et al. 1996; Sereno et al. 1995; Tootell et al. 1996), although a later imaging study showed that only a quarter-field is represented in hV4v (Wilms et al. 2010). However, given that the Euclidean distance between the normalized receptor fingerprints of mV4v and hV4v was the smallest of all interspecies comparisons, it is plausible to consider them homolog areas.

Concluding, we identified and characterized eight receptor architectonically distinct areas in the early visual cortex of the macaque monkey, i.e., V1d, V1v, V2d, V2v, V3d, V3v, V3A and V4v, and compared their fingerprints with those of their homologs in the human brain. Multivariate analyses revealed that although macaque and human early visual areas differ in their molecular architecture, within each species the area-specific differences in receptor fingerprints reflected comparable hierarchical processing levels. Furthermore, in both species the subdivisions of areas V2 and V3 were found to be more closely grouped, i.e., to bear a closer neurochemical resemblance to each other than to remaining areas, and were clearly segregated from the subdivisions of the primary visual cortex and also from V4v. Thus, the macaque monkey early visual cortex can be considered as a good animal model for translational studies.

## Supplementary Information

Below is the link to the electronic supplementary material.

Supplementary file1 (DOCX 4710 KB)

## Data Availability

The authors confirm that the data supporting the findings of this study are available within the article and its supplementary materials. The parcellation scheme and the receptor fingerprints of all examined areas are also made available to the neuroscientific community under https://balsa.wustl.edu/study/l77k6, and the Ebrains platform from the Human Brain Project (https://search.kg.ebrains.eu/instances/Project/e39a0407-a98a-480e-9c63-4a2225ddfbe4).

## Code availability

Original code has been deposited at GitHub: https://github.com/MeiqiNiu/early-visual_human_monkey.
