# Supplementary material for: Receptor architecture of macaque and human early visual areas: not equal, but comparable
Source: Brain Struct Funct. 2021 Dec 20;227(4):1247–63. doi: 10.1007/s00429-021-02437-y (PMC9046358; doi:10.1007/s00429-021-02437-y)
Supplement: Supplementary file 1 — Supplementary file1 (DOCX 4710 KB) [file 429_2021_2437_MOESM1_ESM.docx]

**Supplementary material**

**Receptor architecture of macaque and human early visual areas: not equal, but comparable**

Lucija Rapan^1^*, Meiqi Niu^1^*, Ling Zhao^1^, Thomas Funck^1^, Katrin Amunts^1,2^, Karl Zilles^†^, Nicola Palomero-Gallagher^1,2,3^

**Supplementary Figure 1.** Receptor architecture of macaque primary visual area V1. For details see Fig. 3.


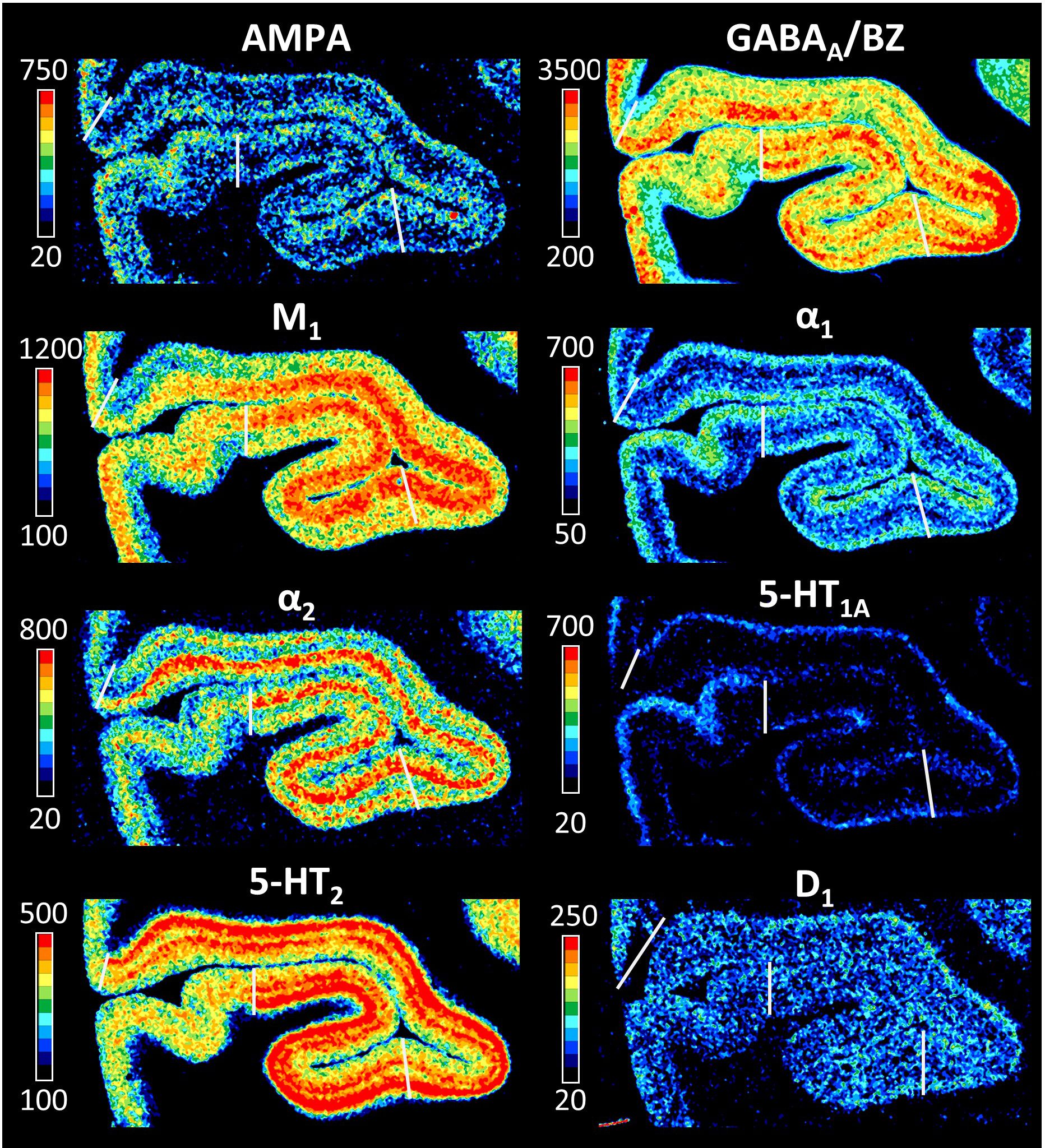


**Supplementary Figure 2.** Receptor architecture of macaque ventral visual areas. For details see Fig. 4.


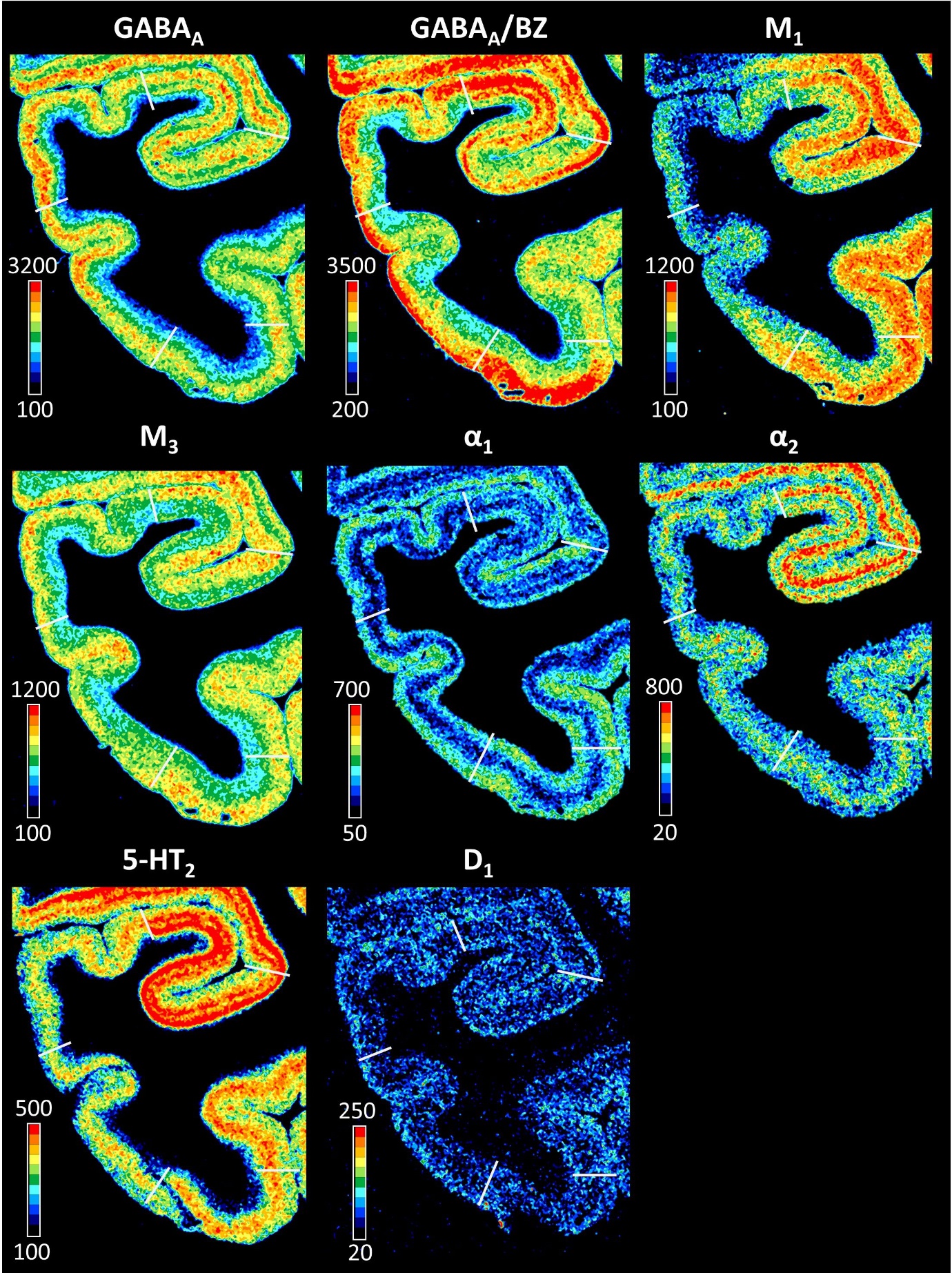


**Supplementary Figure 3.** Receptor architecture of macaque dorsal visual areas. For details see Fig. 5.


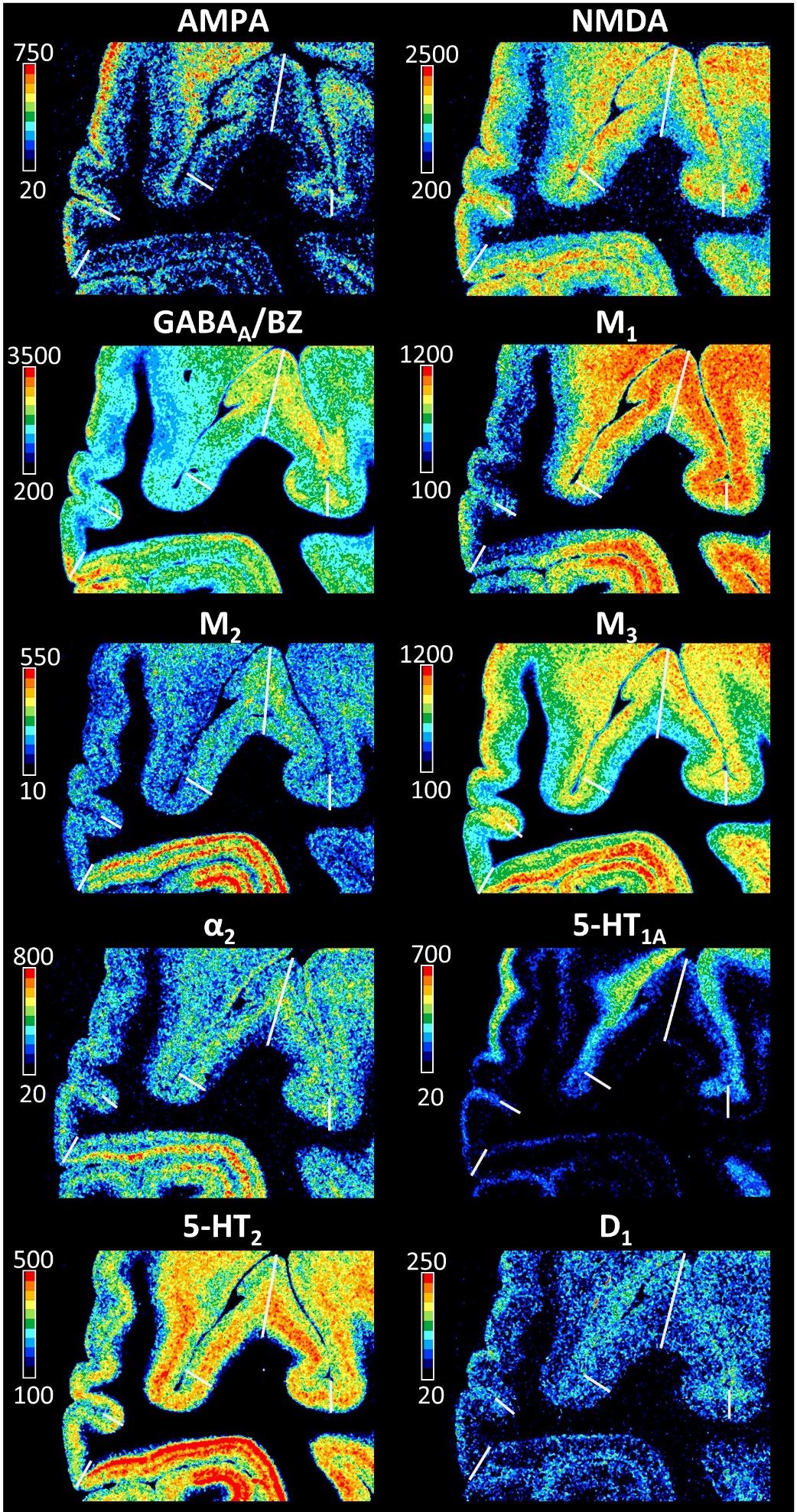


**Supplementary Figure 4.** Schematic representation of the result of the statistical analysis of interareal differences in receptor densities. The receptors highlighted by a coloured background were found to differ significantly between the corresponding pairs of areas, and colour used indicates area with the highest density for that receptor. For details on the statistical strategy see Figure 2, and for statistical values for each level test, see Supplementary Tables 2-5.


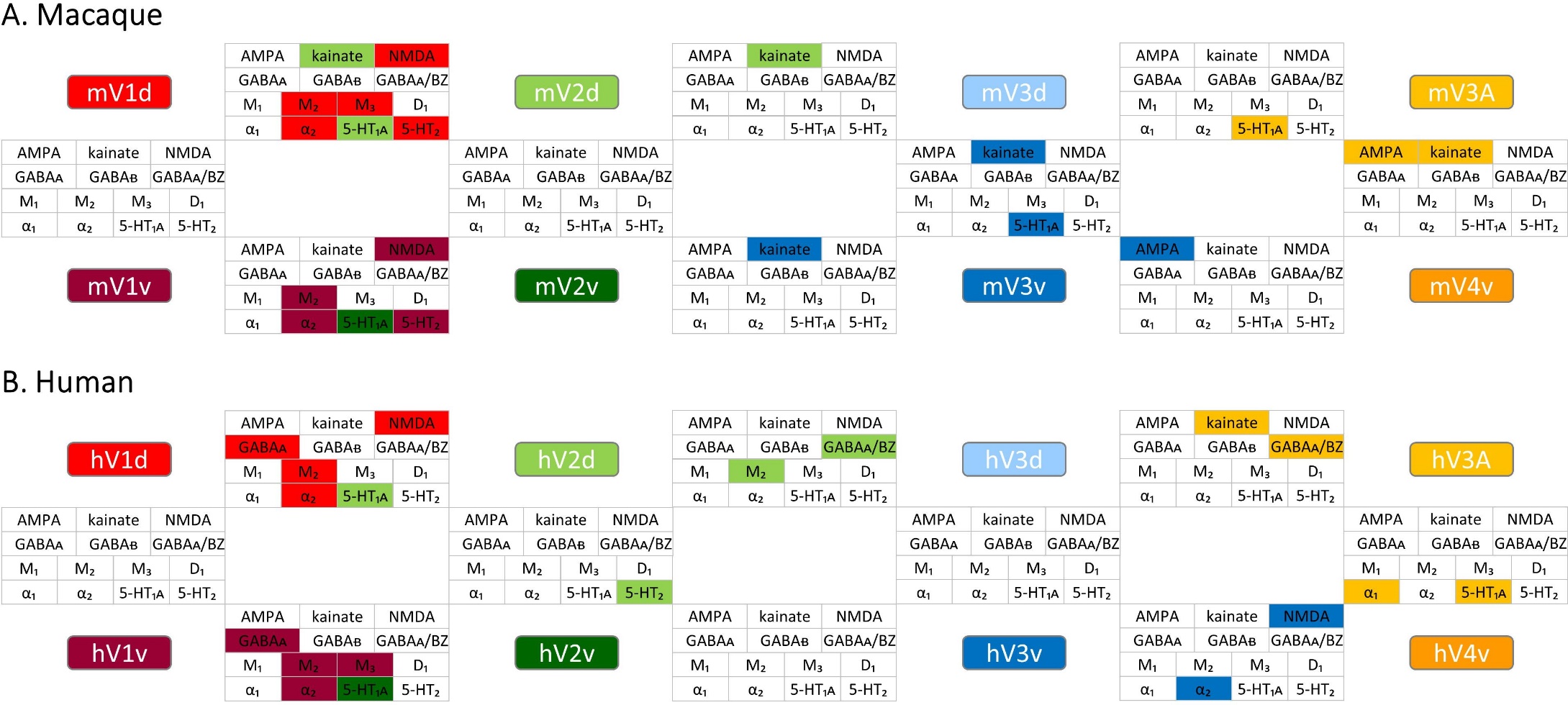


**Supplementary Figure 5.** Schematic representation of the interareal differences in receptor densities between V3v and V3A, as well as those between V3d and V4v. Areas V3v and V3A differed significantly in both the macaque and the human brain. Macaque mV3v contains higher kainate receptor densities than does mV3A. Human hV3v contains higher NMDA and M_1_ but lower kainate and GABA_A_/BZ receptor densities than does hV3A. Areas V3d and V4v differed significantly in both the macaque and the human brain. Macaque mV3d contains lower kainate receptor densities than does mV4v. Human hV3d contains higher NMDA, α_1_ and 5-HT_1A_ but lower GABA_A_/BZ receptor densities than does hV4v. For details on the statistical strategy see Figure 2, and for statistical values for each level test, see Supplementary Tables 2-5.


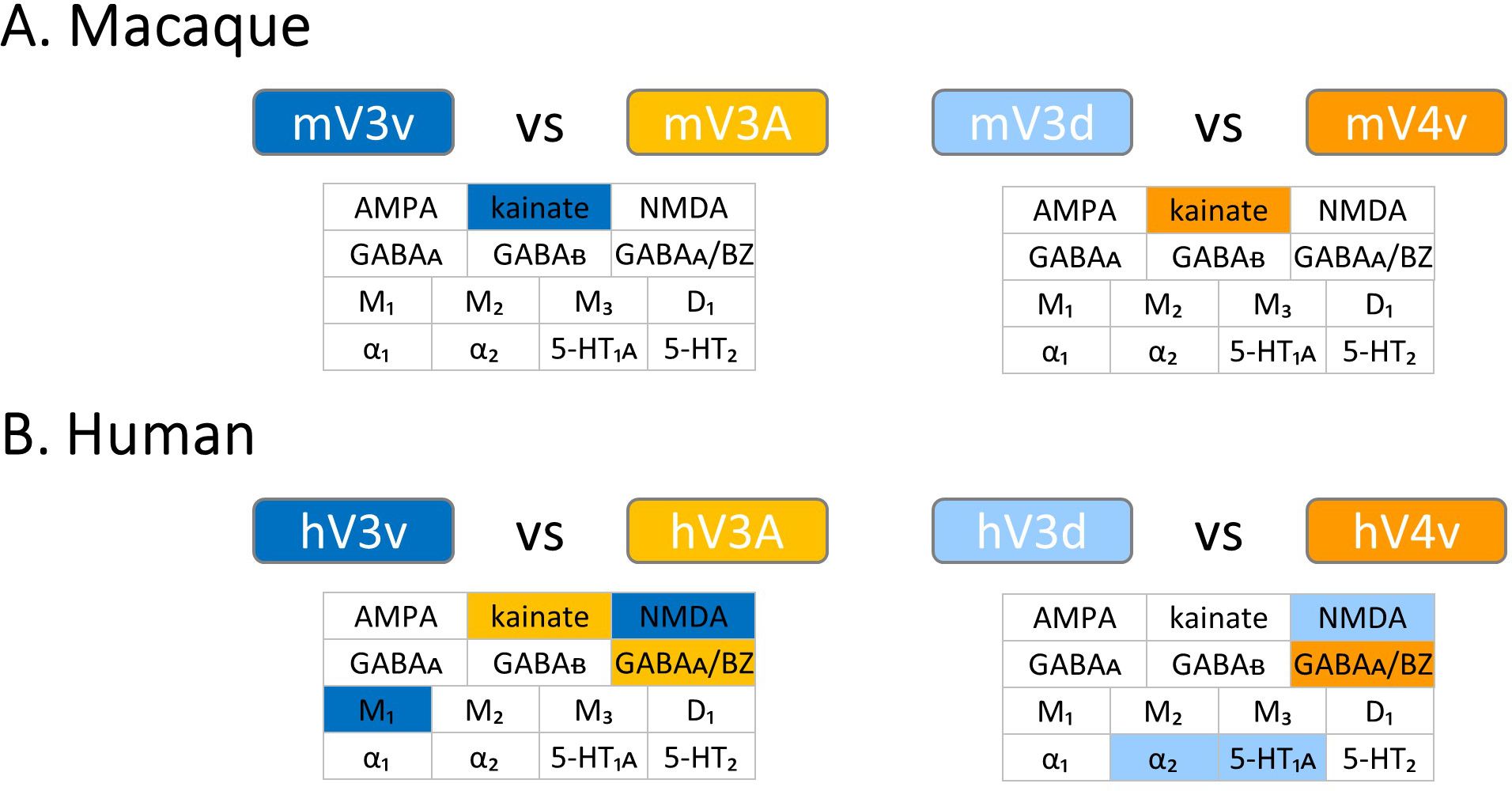


**Supplementary Figure 6.** Normalized receptor fingerprints of the early visual areas in the macaque monkey and human brain. The receptor fingerprints were normalized by min-max scaling, and the thick black line in each plot indicates the average normalized value over all areas. The positions of the different receptor types and the axis scaling (-1.0/+1.0) are identical in all areas, and specified in the polar plot in the middle of the figure.


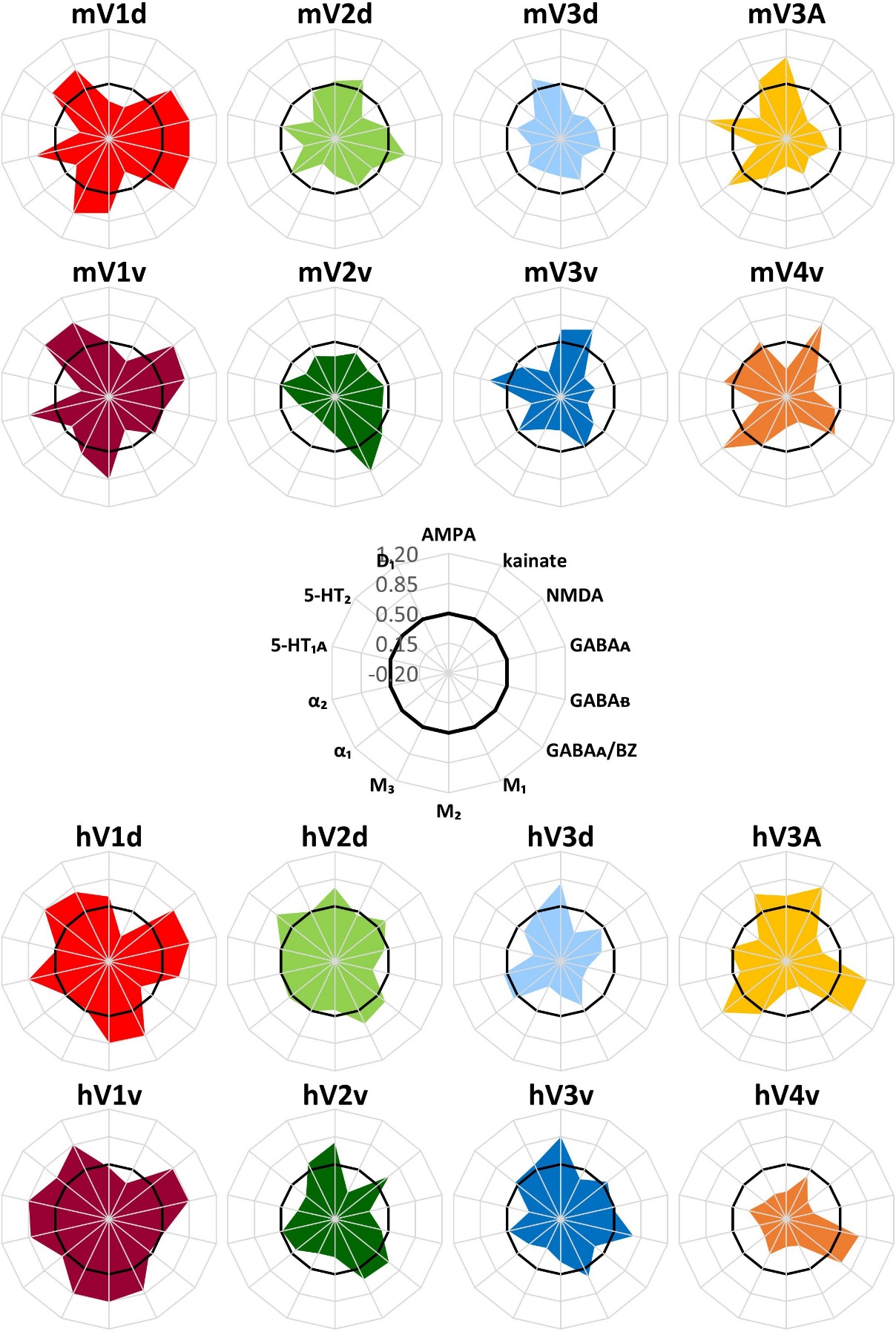


**Supplementary Figure 7.** Elbow plot of the K means analyses aiming to determine the number of stabile clusters of areas of the macaque (red) and human (blue) brains based on (dis)similarities in their normalized receptor fingerprints.

**
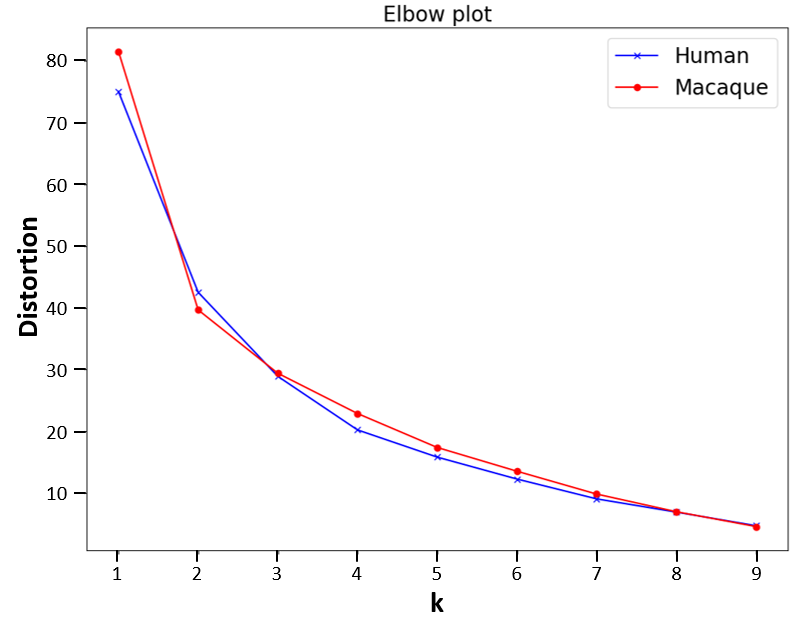
**

**Supplementary Figure 8.** Hierarchical clustering and principal component analysis (PCA) aiming to determine clustering of receptor types based on (dis)similarities in the changes of their densities across visual areas. **A**: Macaque monkey visual areas **B**: Human visual areas. Receptor densities were normalized separately for macaque and human visual areas, and analyses were carried out with a transposed version of the data provided in Supplementary table 7.

**
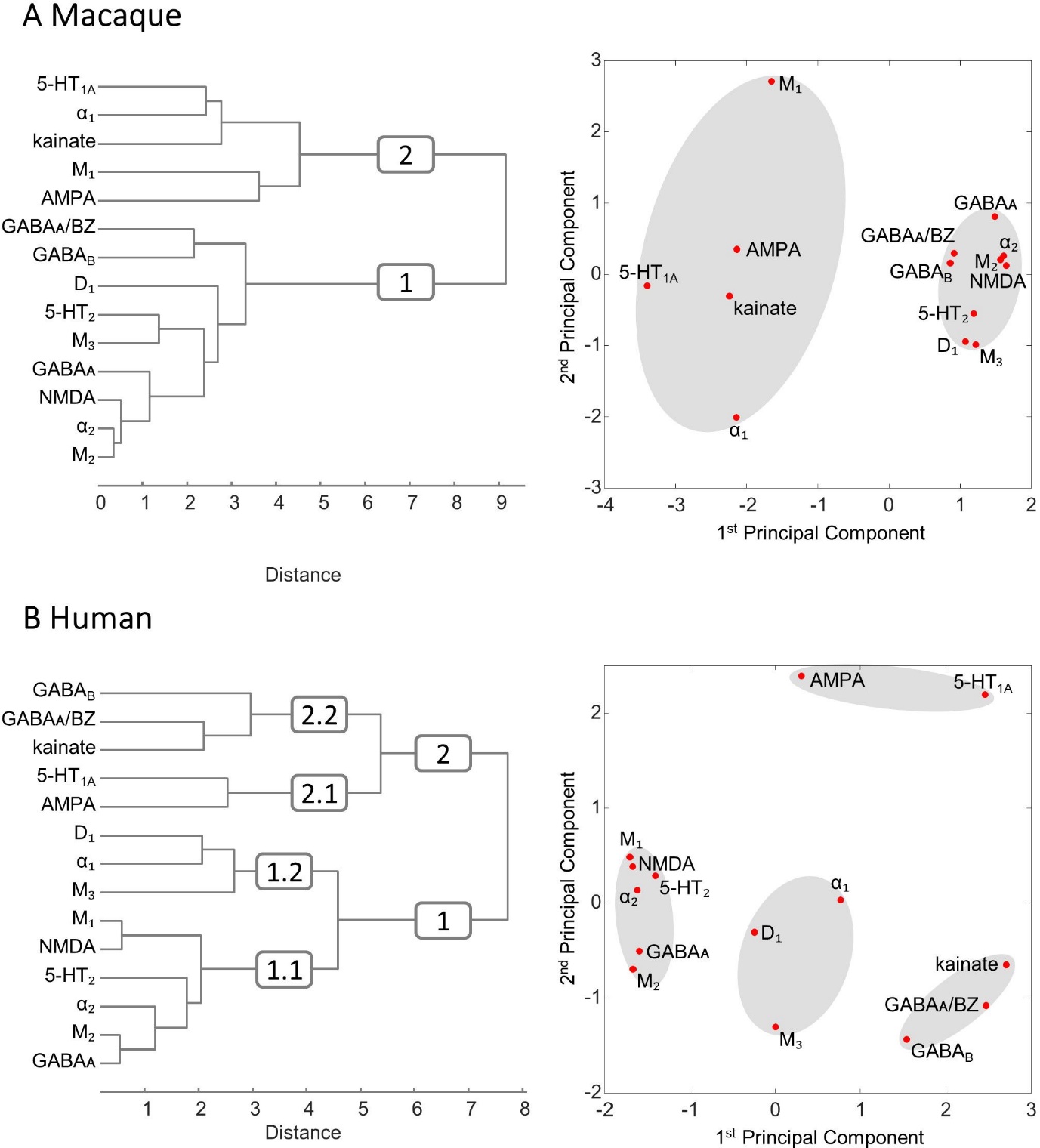
**

**Supplementary Figure 9.** Elbow plot of the K means analyses aiming to determine the number of stabile clusters of receptor types based on (dis)similarities of their expression levels throughout macaque (red) and human (blue) visual areas.

**
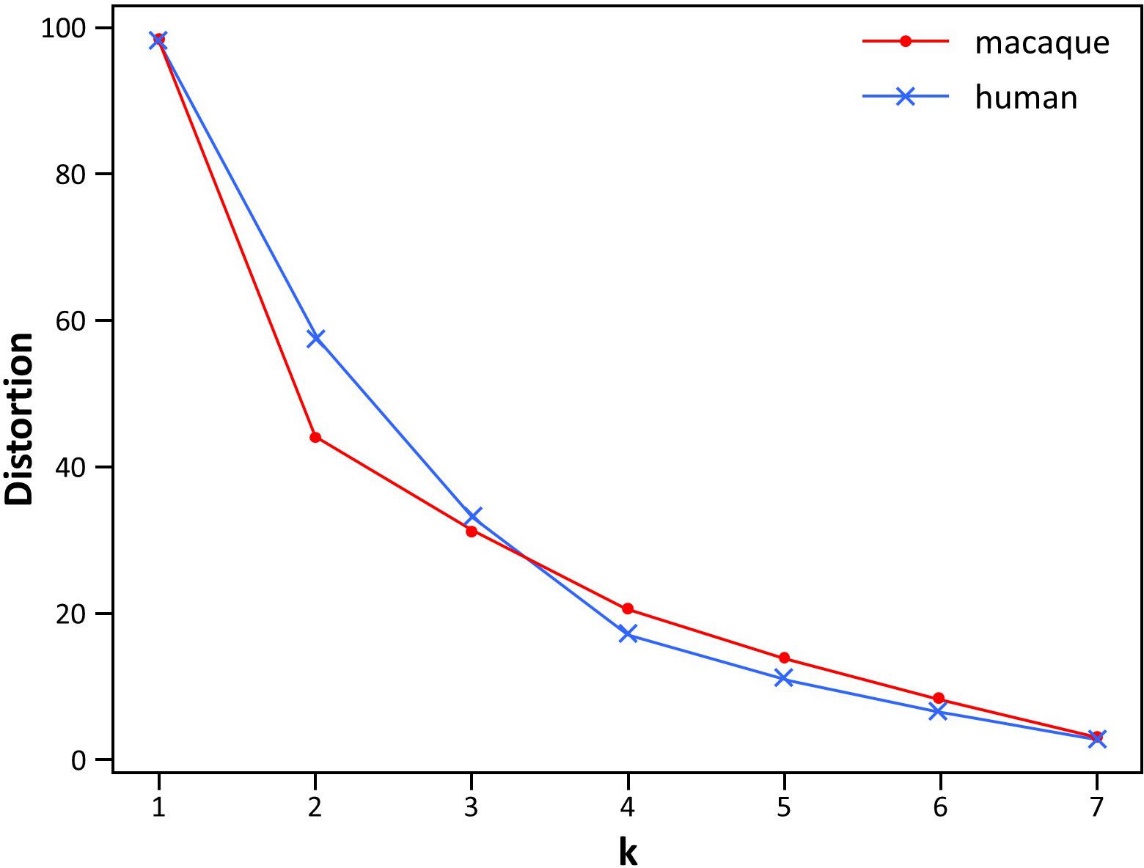
**

**Supplementary Table 1.** Incubation protocols

| Transmitter | Receptor | Ligand (nM) | Displacer | Incubation buffer | Pre-incubation | Main incubation | Final rinsing |
| --- | --- | --- | --- | --- | --- | --- | --- |
| Glutamate | AMPA | [^3^H]-AMPA  (10.0) | Quisqualate  (10 μM) | 50 mM Tris-acetate (pH 7.2) [+ 100 mM KSCN]* | 3x10 min, 4°C | 45 min, 4°C | 1. 4x4 sec 2. Acetone/glutaraldehyde   (100 ml + 2,5 ml), 2x2 sec, 4°C |
|  | NMDA | [^3^H]-MK801  (3.3) | (+)MK-801  (100 μM) | 50 mM Tris-acetate (pH 7.2) + 50 μM glutmate [+ 30 μM glycine + 50 μM spermidine]* | 15 min, 4°C | 60 min, 22°C | 1. 2x5 min, 4°C 2. Distilled water, 1x22°C |
|  | Kainate | [^3^H]-Kainate  (9.4) | SYM 2081  (100 μM) | 50 mM Tris-acetate (pH 7.2) [+ 10 mM Ca^2+^-acetate]* | 3x10 min, 4°C | 45 min, 4°C | 1. 3x4 sec 2. Acetone/glutaraldehyde   (100 ml + 2,5 ml), 2x2 sec, 22°C |
| GABA | GABA_A_ | [^3^H]-Muscimol  (7.7) | GABA  (10 μM) | 50 mM Tris-citrate (pH 7.0) | 3x5 min, 4°C | 40 min, 4°C | 1. 3x3 sec, 4°C 2. Distilled water, 1x22°C |
|  | GABA_B_ | [^3^H]-CGP 54626  (2.0) | CGP 55845  (100 μM) | 50 mM Tris-HCl (pH 7.2) + 2.5 mM CaCl_2_ | 3x5 min, 4°C | 60 min, 4°C | 1. 3x2 sec, 4°C 2. Distilled water, 1x22°C |
|  | GABA_A_/BZ | [^3^H]-Flumazenil  (1.0) | Clonazepam  (2 μM) | 170 mM Tris-HCl (pH 7.4) | 15 min, 4°C | 60 min, 4°C | 1. 2x1 min, 4°C 2. Distilled water, 1x22°C |
| Acetylcholine | M_1_ | [^3^H]-Pirenzepine  (1.0) | Pirenzepine  (2 μM) | Modified Krebs buffer (pH 7.4) | 15 min, 4°C | 60 min, 4°C | 1. 2x1 min, 4°C 2. Distilled water, 1x22°C |
|  | M_2_ | [^3^H]-Oxotremorine-M (1.7) | Carbacol  (10 μM) | 20 mM HEPES-Tris (pH 7.5) + 10 mM MgCl_2_ + 300 nM Pirenzepine | 20 min, 22°C | 60 min, 22°C | 1. 2x2 min, 4°C 2. Distilled water, 1x22°C |
|  | M_3_ | [^3^H]-4-DAMP  (1.0) | Atropine sulfate  (10 μM) | 50 mM Tris-HCl (pH 7.4) + 0.1 mM PSMF + 1mM EDTA | 15 min, 22° C | 45 min, 22° C | 1. 2x5 min, 4° C 2. distilled water, 1x22°C |
| Noradrenaline | α_1_ | [^3^H]-Prazosin  (0.2) | Phentolamine Mesylate  (10 μM) | 50 mM Na/K-phosphate buffer (pH 7.4) | 15 min, 22°C | 60 min, 22°C | 1. 2x5 min, 4°C 2. Distilled water, 1x22°C |
|  | α_2_ | [^3^H]-UK 14,304  (0,64) | Phentolamine Mesylate  (10 μM) | 50 mM Tris-HCl + 100 μM MnCl_2_ (pH 7.7) | 15 min, 22°C | 90 min, 22°C | 1. 5 min, 4°C 2. Distilled water, 1x22°C |
| Serotonin | 5-HT_1A_ | [^3^H]-8-OH-DPAT  (1.0) | 5-Hydroxy-tryptamine  (1 μM) | 170 mM Tris-HCl (pH 7.4) [+ 4 mM CaCl_2_ + 0.01% ascorbate]* | 30 min, 22°C | 60 min, 22°C | 1. 5 min, 4°C 2. Distilled water, 3x22°C |
|  | 5-HT_2_ | [^3^H]-Ketanserin  (1.14) | Mianserin  (10 μM) | 170 mM Tris-HCl (pH 7.7) | 30 min, 22°C | 120 min, 22°C | 1. 2x10 min, 4°C 2. Distilled water, 3x22°C |
| Dopamine | D_1_ | [^3^H]-SCH 23390  (1.67) | SKF 83566  (1 μM) | 50 mM Tris-HCl + 120 mM NaCl + 5 mM KCl + 2 mM CaCl_2_ + 1 mM MgCl_2_ (pH 7.4) | 20 min, 22°C | 90 min, 22°C | 1. 2x20 min, 4°C 2. Distilled water, 1x22°C |
| Adenosine | A_1_ | [^3^H]-DPCPX  (1.0) | R-PIA  (100 μM) | 170 mM Tris-HCl + 2 Units/I Adenosine deaminase [+ 100 μM Gpp(NH)p]* (pH 7.4) | 15 min, 4°C | 120 min, 22°C | 1. 2x5 min, 4°C 2. Distilled water, 1x22°C |

***** Substances in square brackets only included in the main incubation

**Supplementary Table 2.** Statistic values (e.g., F value and *p* value) for the omnibus test (i.e., the first level test) of macaque and human early visual areas.

|  | Macaque | |  | Human | |
| --- | --- | --- | --- | --- | --- |
|  | F value | *p* value |  | F value | *p* value |
| Area | 11.763 | 1.56E-14*** |  | 17.2632 | 2.20E-16*** |
| Receptor | 33.801 | 2.20E-16*** |  | 33.1286 | 2.20E-16*** |
| Area × Receptor | 4.62 | 2.20E-16*** |  | 5.9207 | 2.20E-16*** |

⁎ *p* < 0.05, ⁎⁎ *p* < 0.01, ⁎⁎⁎ *p* < 0.001

**Supplementary Table 3.** Statistic values (e.g., F value and corrected *p* value) for the simple effect tests (i.e., the second level test) of macaque and human early visual areas.

|  | Macaque | |  | Human | |
| --- | --- | --- | --- | --- | --- |
|  | F value | *p_*corr |  | F value | *p*_corr |
| AMPA | 3.262 | 0.0035** |  | 2.24 | 0.0383* |
| kainate | 8.452 | 0.0004*** |  | 4.267 | 0.0002*** |
| NMDA | 3.862 | 0.0009*** |  | 9.365 | 0.0002*** |
| GABA_A_ | 3.79 | 0.0009*** |  | 7.246 | 0.0002*** |
| GABA_B_ | 2.258 | 0.0323* |  | 0.949 | 0.5032 |
| GABA_A_/BZ | 3.735 | 0.0010** |  | 4.126 | 0.0004*** |
| M_1_ | 1.29 | 0.2517 |  | 5.289 | 0.0002*** |
| M_2_ | 11.085 | 0.0004*** |  | 27.054 | 0.0002*** |
| M_3_ | 2.739 | 0.0112* |  | 2.126 | 0.0443* |
| α_1_ | 2.543 | 0.0171** |  | 2.22 | 0.0383* |
| α_2_ | 17.587 | 0.0004*** |  | 13.795 | 0.0002*** |
| 5-HT_1A_ | 6.896 | 0.0004*** |  | 12.354 | 0.0002*** |
| 5-HT_2_ | 2.928 | 0.0075** |  | 3.217 | 0.0032** |
| D_1_ | 1.677 | 0.1193 |  | 0.802 | 0.5859 |

⁎ *p* < 0.05, ⁎⁎ *p* < 0.01, ⁎⁎⁎ *p* < 0.001

**Supplementary Table 4.** FDR-corrected *p*-values for the post hoc tests (i.e., third level tests) of macaque early visual areas. Correction was carried out for each receptor type separately (i.e., *p*-values were corrected for 28 comparisons per receptor type). Note, that no *p*-values are provided for the M_1_ or D_1_ receptors, because they did not reach the level of significance in the second level test.

| Macaque | AMPA | kainate | NMDA | GABAᴀ | GABAᴃ | GABAᴀ/BZ | M₂ | M₃ | α₁ | α₂ | 5-HT₁ᴀ | 5-HT₂ |
| --- | --- | --- | --- | --- | --- | --- | --- | --- | --- | --- | --- | --- |
| mV1d/mV1v | 0.4234 | 0.7081 | 0.8546 | 0.8829 | 0.3327 | 0.0905 | 0.6412 | 0.4687 | 0.8008 | 0.6568 | 0.8803 | 0.7422 |
| mV1d/mV2d | 0.2893 | 0.0055** | 0.0165* | 0.1224 | 0.7651 | 0.0905 | 0.0001*** | 0.0134* | 0.5152 | 0.0001*** | 0.0377* | 0.035* |
| mV1d/mV2v | 0.8509 | 0.2525 | 0.0461* | 0.0986 | 0.2255 | 0.3029 | 0.0001*** | 0.0134* | 0.5635 | 0.0001*** | 0.0274* | 0.1005 |
| mV1d/mV3A | 0.0148* | 0.8317 | 0.0108* | 0.0118* | 0.1207 | 0.0017** | 0.0001*** | 0.0799 | 0.1564 | 0.0001*** | 0.0001*** | 0.0727 |
| mV1d/mV3d | 0.4234 | 0.5144 | 0.0165* | 0.0135* | 0.1207 | 0.0017** | 0.0001*** | 0.0343* | 0.9845 | 0.0001*** | 0.1852 | 0.0727 |
| mV1d/mV3v | 0.1301 | 0.0003*** | 0.011* | 0.0118* | 0.0276* | 0.0102* | 0.0001*** | 0.0343* | 0.6063 | 0.0001*** | 0.0002*** | 0.4187 |
| mV1d/mV4v | 0.5632 | 0.0001*** | 0.0272* | 0.0066** | 0.2255 | 0.1062 | 0.0001*** | 0.2689 | 0.0868 | 0.0001*** | 0.0032** | 0.438 |
| mV1v/mV2d | 0.8211 | 0.0186* | 0.011* | 0.2046 | 0.5907 | 0.6953 | 0.0001*** | 0.1255 | 0.6299 | 0.0001*** | 0.0274* | 0.028* |
| mV1v/mV2v | 0.4397 | 0.4315 | 0.0272* | 0.1677 | 0.7651 | 0.7289 | 0.0001*** | 0.1362 | 0.4451 | 0.0001*** | 0.0228* | 0.035* |
| mV1v/mV3A | 0.1301 | 0.8317 | 0.0108* | 0.0184* | 0.6056 | 0.1062 | 0.0001*** | 0.4445 | 0.2308 | 0.0001*** | 0.0001*** | 0.0327* |
| mV1v/mV3d | 0.9799 | 0.3314 | 0.011* | 0.0225* | 0.5907 | 0.0916 | 0.0001*** | 0.29 | 0.8008 | 0.0001*** | 0.14 | 0.0327* |
| mV1v/mV3v | 0.4397 | 0.0012** | 0.0108* | 0.0185* | 0.2542 | 0.368 | 0.0001*** | 0.2851 | 0.7864 | 0.0001*** | 0.0001*** | 0.2235 |
| mV1v/mV4v | 0.145 | 0.0001*** | 0.0165* | 0.0105* | 0.7651 | 0.8261 | 0.0001*** | 0.7432 | 0.1564 | 0.0001*** | 0.0022** | 0.2451 |
| mV2d/mV2v | 0.4077 | 0.1549 | 0.8546 | 0.9481 | 0.3801 | 0.4878 | 0.9599 | 0.9528 | 0.2005 | 0.8017 | 0.8861 | 0.7422 |
| mV2d/mV3A | 0.1743 | 0.01* | 0.8546 | 0.445 | 0.2542 | 0.3414 | 0.539 | 0.5624 | 0.5152 | 0.9482 | 0.0231* | 0.8629 |
| mV2d/mV3d | 0.8211 | 0.0006*** | 0.9984 | 0.47 | 0.2377 | 0.3029 | 0.9599 | 0.7432 | 0.5152 | 0.9482 | 0.4531 | 0.875 |
| mV2d/mV3v | 0.5864 | 0.4315 | 0.8546 | 0.4535 | 0.0958 | 0.7289 | 0.8312 | 0.7522 | 0.8227 | 0.9482 | 0.1152 | 0.3605 |
| mV2d/mV4v | 0.1301 | 0.1437 | 0.8546 | 0.2052 | 0.3897 | 0.5568 | 0.6897 | 0.3031 | 0.3156 | 0.8746 | 0.4111 | 0.2857 |
| mV2v/mV3A | 0.0215* | 0.3314 | 0.7098 | 0.47 | 0.7838 | 0.0916 | 0.4968 | 0.6085 | 0.0441* | 0.6568 | 0.0274* | 0.927 |
| mV2v/mV3d | 0.4397 | 0.061 | 0.8546 | 0.5236 | 0.7651 | 0.0905 | 0.9883 | 0.7522 | 0.5635 | 0.6568 | 0.4111 | 0.9161 |
| mV2v/mV3v | 0.145 | 0.0188* | 0.8381 | 0.47 | 0.4112 | 0.3029 | 0.8198 | 0.7522 | 0.2572 | 0.6568 | 0.14 | 0.5247 |
| mV2v/mV4v | 0.4397 | 0.0018** | 0.8546 | 0.2488 | 0.952 | 0.8261 | 0.6777 | 0.3322 | 0.0197* | 0.5092 | 0.4531 | 0.4397 |
| mV3A/mV3d | 0.1301 | 0.4315 | 0.8546 | 0.9481 | 0.952 | 0.9193 | 0.4968 | 0.7703 | 0.1564 | 0.9736 | 0.0023** | 0.9563 |
| mV3A/mV3v | 0.4377 | 0.0006*** | 0.8546 | 0.9596 | 0.5907 | 0.4878 | 0.6897 | 0.7522 | 0.4047 | 0.9736 | 0.4534 | 0.4397 |
| mV3A/mV4v | 0.0018** | 0.0001*** | 0.8381 | 0.76 | 0.7651 | 0.0905 | 0.8312 | 0.7432 | 0.7864 | 0.9482 | 0.1496 | 0.4187 |
| mV3d/mV3v | 0.4397 | 0.0001*** | 0.8546 | 0.9481 | 0.6056 | 0.4406 | 0.8198 | 0.9528 | 0.6063 | 0.9736 | 0.0228* | 0.438 |
| mV3d/mV4v | 0.145 | 0.0001*** | 0.8546 | 0.6892 | 0.7651 | 0.0905 | 0.6777 | 0.5624 | 0.0868 | 0.9482 | 0.1152 | 0.4187 |
| mV3v/mV4v | 0.0263* | 0.4852 | 0.8546 | 0.7495 | 0.3982 | 0.3029 | 0.8408 | 0.5541 | 0.2308 | 0.9482 | 0.4531 | 0.9302 |

Pink background highlights pairs of adjacent early visual areas in the macaque brain. ⁎ *p* < 0.05, ⁎⁎ *p* < 0.01, ⁎⁎⁎ *p* < 0.001

**Supplementary Table 5.** FDR-corrected *p*-values for the post hoc tests (i.e., third level tests) of human early visual areas. (i.e., *p*-values were corrected for 28 comparisons per receptor type). Note, that no *p*-values are provided for the GABA_B_ or D_1_ receptors, because they did not reach the level of significance in the second level test.

| Human | AMPA | kainate | NMDA | GABAᴀ | GABAᴀ/BZ | M₁ | M₂ | M₃ | α₁ | α₂ | 5-HT₁ᴀ | 5-HT₂ |
| --- | --- | --- | --- | --- | --- | --- | --- | --- | --- | --- | --- | --- |
| hV1d/hV1v | 0.4636 | 0.4866 | 0.8219 | 0.5618 | 0.2268 | 0.7741 | 0.908 | 0.4081 | 0.6365 | 0.9919 | 0.2632 | 0.3822 |
| hV1d/hV2d | 0.5712 | 0.0763 | 0.0299* | 0.0027** | 0.2317 | 0.089 | 0.0001*** | 0.9411 | 0.7442 | 0.0001*** | 0.0004*** | 0.7705 |
| hV1d/hV2v | 0.5712 | 0.8912 | 0.1249 | 0.0001*** | 0.0874 | 0.2536 | 0.0001*** | 0.4479 | 0.8992 | 0.0001*** | 0.0069** | 0.0111* |
| hV1d/hV3A | 0.9675 | 0.0003 | 0.0001*** | 0.0001*** | 0.0115* | 0.0002*** | 0.0001*** | 0.9411 | 0.1968 | 0.0001*** | 0.0001*** | 0.0111* |
| hV1d/hV3d | 0.5712 | 0.8694 | 0.001** | 0.0001*** | 0.3401 | 0.0037** | 0.0001*** | 0.4081 | 0.7643 | 0.0001*** | 0.0001*** | 0.1538 |
| hV1d/hV3v | 0.5712 | 0.3856 | 0.0538 | 0.0027** | 0.8512 | 0.1501 | 0.0001*** | 0.4081 | 0.6365 | 0.0001*** | 0.0001*** | 0.3514 |
| hV1d/hV4v | 0.1537 | 0.1756 | 0.0001*** | 0.0001*** | 0.0681 | 0.0014** | 0.0001*** | 0.4479 | 0.2427 | 0.0001*** | 0.0842 | 0.1538 |
| hV1v/hV2d | 0.1175 | 0.3134 | 0.0538 | 0.0159* | 0.9593 | 0.1793 | 0.0001*** | 0.3006 | 0.8206 | 0.0001*** | 0.0001*** | 0.5244 |
| hV1v/hV2v | 0.1091 | 0.5833 | 0.201 | 0.0001*** | 0.6296 | 0.4278 | 0.0001*** | 0.0388* | 0.547 | 0.0001*** | 0.0001*** | 0.1289 |
| hV1v/hV3A | 0.4636 | 0.0045** | 0.0001*** | 0.0002*** | 0.2268 | 0.0009*** | 0.0001*** | 0.373 | 0.4167 | 0.0001*** | 0.0001*** | 0.1003 |
| hV1v/hV3d | 0.1091 | 0.6148 | 0.0019** | 0.0008*** | 0.0222* | 0.0119* | 0.0001*** | 0.0388* | 0.8014 | 0.0001*** | 0.0001*** | 0.5244 |
| hV1v/hV3v | 0.1091 | 0.8398 | 0.0832 | 0.0156* | 0.3401 | 0.2575 | 0.0001*** | 0.0388* | 0.2952 | 0.0001*** | 0.0001*** | 0.8535 |
| hV1v/hV4v | 0.5795 | 0.4866 | 0.0001*** | 0.0002*** | 0.3872 | 0.0046** | 0.0001*** | 0.0618 | 0.1082 | 0.0001*** | 0.0094** | 0.5098 |
| hV2d/hV2v | 0.9675 | 0.0957 | 0.5396 | 0.2066 | 0.6296 | 0.5948 | 0.1302 | 0.5898 | 0.6784 | 0.71 | 0.442 | 0.0219* |
| hV2d/hV3A | 0.5795 | 0.1054 | 0.0023** | 0.2123 | 0.2268 | 0.0295* | 0.0017** | 0.9411 | 0.2952 | 0.3 | 0.218 | 0.0176* |
| hV2d/hV3d | 0.9675 | 0.0957 | 0.2649 | 0.4715 | 0.0274* | 0.229 | 0.0112 | 0.4839 | 0.9498 | 0.7593 | 0.0766 | 0.2595 |
| hV2d/hV3v | 0.9675 | 0.4866 | 0.8553 | 0.9285 | 0.3649 | 0.7859 | 0.1262 | 0.4839 | 0.4167 | 0.6477 | 0.3511 | 0.5098 |
| hV2d/hV4v | 0.054 | 0.8398 | 0.0012** | 0.1575 | 0.3872 | 0.0935 | 0.014* | 0.5898 | 0.1388 | 0.0029** | 0.3065 | 0.2595 |
| hV2v/hV3A | 0.5712 | 0.0003*** | 0.0003*** | 0.9908 | 0.4126 | 0.007** | 0.1748 | 0.4839 | 0.149 | 0.5293 | 0.0456* | 0.8535 |
| hV2v/hV3d | 0.9675 | 0.9446 | 0.0765 | 0.6471 | 0.0083** | 0.0855 | 0.435 | 0.9411 | 0.6784 | 0.5144 | 0.0094** | 0.3582 |
| hV2v/hV3v | 0.9675 | 0.4671 | 0.6459 | 0.2627 | 0.2011 | 0.7741 | 0.9673 | 0.9411 | 0.6784 | 0.9417 | 0.0842 | 0.1538 |
| hV2v/hV4v | 0.054 | 0.2174 | 0.0002*** | 0.7283 | 0.6296 | 0.0295* | 0.4052 | 0.9411 | 0.2869 | 0.0095** | 0.6678 | 0.5244 |
| hV3A/hV3d | 0.5712 | 0.0003*** | 0.0569 | 0.6471 | 0.0004*** | 0.3459 | 0.5759 | 0.4479 | 0.2952 | 0.1483 | 0.6678 | 0.3014 |
| hV3A/hV3v | 0.5712 | 0.0187* | 0.002** | 0.2651 | 0.0329* | 0.0181* | 0.1797 | 0.4479 | 0.108 | 0.5893 | 0.7172 | 0.1498 |
| hV3A/hV4v | 0.1537 | 0.0957 | 0.6184 | 0.7283 | 0.8575 | 0.7741 | 0.7695 | 0.4839 | 0.0126* | 0.0648 | 0.0437* | 0.5098 |
| hV3d/hV3v | 0.9675 | 0.4866 | 0.2176 | 0.5438 | 0.2806 | 0.1551 | 0.4425 | 0.9411 | 0.4167 | 0.4561 | 0.442 | 0.6207 |
| hV3d/hV4v | 0.054 | 0.2342 | 0.0267* | 0.4801 | 0.0083** | 0.5937 | 0.8931 | 0.9488 | 0.1388 | 0.0008*** | 0.0121* | 0.8535 |
| hV3v/hV4v | 0.054 | 0.6725 | 0.0011** | 0.205 | 0.1298 | 0.0686 | 0.4092 | 0.9411 | 0.4951 | 0.0121* | 0.0766 | 0.5436 |

Pink background highlights pairs of adjacent early visual areas in the human brain. ⁎ *p* < 0.05, ⁎⁎ *p* < 0.01, ⁎⁎⁎ *p* < 0.001

**Supplementary Table 6.** Absolute receptor densities (mean±sd in fmol/mg protein) of macaque and human early visual areas. Cells have been color-coded to facilitate identification of areas with higher (warm tones) and lower (cool tones) receptor densities.

|  | AMPA | kainate | NMDA | GABAᴀ | GABAᴃ | GABAᴀ/BZ | M₁ | M₂ | M₃ | α₁ | α₂ | 5-HT₁ᴀ | 5-HT₂ | D₁ |
| --- | --- | --- | --- | --- | --- | --- | --- | --- | --- | --- | --- | --- | --- | --- |
| Macaque |  |  |  |  |  |  |  |  |  |  |  |  |  |  |
| mV1d | 285±34 | 483±38 | 1379±73 | 1889±268 | 1855±242 | 2344±305 | 865±352 | 342±82 | 840±109 | 249±55 | 466±24 | 63±25 | 404±80 | 78±11 |
| mV1v | 319±25 | 502±50 | 1392±54 | 1847±243 | 1723±252 | 2038±187 | 865±348 | 377±96 | 792±121 | 257±48 | 493±21 | 60±26 | 421±96 | 80±15 |
| mV2d | 327±67 | 613±29 | 1214±110 | 1615±362 | 1805±130 | 1889±13 | 940±227 | 175±45 | 704±86 | 271±51 | 300±12 | 102±21 | 321±83 | 70±4 |
| mV2v | 291±28 | 542±34 | 1242±53 | 1597±259 | 1682±206 | 2062±165 | 1078±22 | 179±54 | 708±67 | 231±53 | 319±44 | 104±31 | 338±77 | 68±5 |
| mV3A | 378±30 | 492±50 | 1187±30 | 1455±269 | 1653±275 | 1647±3 | 879±111 | 133±23 | 742±97 | 294±59 | 292±46 | 145±44 | 333±70 | 74±10 |
| mV3d | 318±46 | 452±38 | 1214±81 | 1477±378 | 1645±256 | 1628±19 | 907±87 | 179±60 | 728±69 | 250±41 | 295±26 | 88±34 | 331±72 | 75±5 |
| mV3v | 346±75 | 652±53 | 1197±119 | 1462±343 | 1575±174 | 1823±255 | 954±281 | 161±55 | 725±93 | 266±60 | 294±48 | 132±43 | 359±84 | 62±9 |
| mV4v | 264±60 | 686±80 | 1229±104 | 1389±388 | 1688±214 | 2073±363 | 823±332 | 148±46 | 765±104 | 303±46 | 285±63 | 118±34 | 369±73 | 73±21 |
| Human |  |  |  |  |  |  |  |  |  |  |  |  |  |  |
| hV1d | 279±66 | 319±58 | 1484±317 | 2356±642 | 1757±177 | 1746±260 | 516±129 | 359±75 | 1004±424 | 339±86 | 853±180 | 149±47 | 344±48 | 104±23 |
| hV1v | 245±39 | 360±115 | 1474±305 | 2347±773 | 1686±176 | 1913±380 | 503±134 | 361±66 | 1204±359 | 351±102 | 830±208 | 127±23 | 331±28 | 106±23 |
| hV2d | 304±73 | 400±108 | 1314±250 | 1842±520 | 1595±250 | 2102±590 | 470±158 | 236±38 | 1017±187 | 349±100 | 583±106 | 206±45 | 338±62 | 97±24 |
| hV2v | 312±90 | 325±64 | 1352±289 | 1593±433 | 1640±340 | 2168±821 | 462±93 | 194±64 | 899±321 | 327±96 | 563±177 | 203±57 | 306±45 | 99±14 |
| hV3A | 281±79 | 509±246 | 1051±444 | 1624±662 | 1813±531 | 2368±921 | 327±63 | 162±57 | 1041±317 | 406±147 | 501±262 | 223±64 | 305±60 | 103±28 |
| hV3d | 315±109 | 332±59 | 1187±312 | 1704±653 | 1537±229 | 1548±256 | 401±108 | 184±46 | 827±271 | 351±91 | 613±107 | 238±70 | 315±50 | 97±22 |
| hV3v | 327±110 | 377±82 | 1266±395 | 1875±602 | 1773±339 | 1783±283 | 451±112 | 214±97 | 859±263 | 304±82 | 557±100 | 228±60 | 324±49 | 98±27 |
| hV4v | 181±60 | 390±119 | 945±404 | 1467±565 | 1772±633 | 2181±904 | 332±85 | 155±42 | 904±143 | 274±116 | 299±144 | 184±51 | 298±69 | 87±22 |

**Supplementary Table 7.** Z-score normalized receptor densities used for cluster analyses depicted in Figure 8. Note, that for each receptor type normalization was carried out separately for macaque and human areas. The datasets were transposed to carry out the cluster analyses shown in Supplementary Fig. 8. Cells have been color-coded to facilitate identification of areas with higher (warm tones) and lower (cool tones) densities than the average for the respective receptor type.

|  | AMPA | kainate | NMDA | GABAᴀ | GABAᴃ | GABAᴀ/BZ | M₁ | M₂ | M₃ | α₁ | α₂ | 5-HT₁ᴀ | 5-HT₂ | D₁ |
| --- | --- | --- | --- | --- | --- | --- | --- | --- | --- | --- | --- | --- | --- | --- |
| Macaque |  |  |  |  |  |  |  |  |  |  |  |  |  |  |
| mV1d | -0.03 | -0.94 | 1.18 | 1.50 | 0.62 | -0.84 | 1.15 | 1.52 | 0.28 | 0.03 | 1.42 | -1.16 | 1.43 | 0.87 |
| mV1v | -0.73 | -0.26 | 1.12 | 1.48 | -0.10 | -0.23 | 0.97 | 1.55 | 1.91 | 0.34 | 1.30 | -1.73 | 0.65 | 1.20 |
| mV2d | 0.49 | 0.38 | 0.29 | -0.03 | -1.03 | 0.46 | 0.52 | 0.03 | 0.39 | 0.29 | -0.09 | 0.29 | 1.05 | -0.39 |
| mV2v | 0.65 | -0.83 | 0.49 | -0.77 | -0.58 | 0.70 | 0.40 | -0.47 | -0.58 | -0.26 | -0.21 | 0.20 | -0.85 | 0.01 |
| mV3A | 0.02 | 2.15 | -1.09 | -0.68 | 1.18 | 1.43 | -1.46 | -0.86 | 0.59 | 1.76 | -0.56 | 0.71 | -0.92 | 0.75 |
| mV3d | 0.71 | -0.73 | -0.38 | -0.44 | -1.62 | -1.56 | -0.44 | -0.60 | -1.16 | 0.36 | 0.07 | 1.12 | -0.31 | -0.37 |
| mV3v | 0.97 | 0.01 | 0.03 | 0.07 | 0.77 | -0.70 | 0.25 | -0.24 | -0.90 | -0.87 | -0.24 | 0.84 | 0.24 | -0.08 |
| mV4v | -2.08 | 0.21 | -1.64 | -1.14 | 0.77 | 0.75 | -1.39 | -0.94 | -0.54 | -1.65 | -1.69 | -0.28 | -1.30 | -1.99 |
| Human |  |  |  |  |  |  |  |  |  |  |  |  |  |  |
| hV1d | -0.03 | -0.94 | 1.18 | 1.50 | 0.62 | -0.84 | 1.15 | 1.52 | 0.28 | 0.03 | 1.42 | -1.16 | 1.43 | 0.87 |
| hV1v | -0.73 | -0.26 | 1.12 | 1.48 | -0.10 | -0.23 | 0.97 | 1.55 | 1.91 | 0.34 | 1.30 | -1.73 | 0.65 | 1.20 |
| hV2d | 0.49 | 0.38 | 0.29 | -0.03 | -1.03 | 0.46 | 0.52 | 0.03 | 0.39 | 0.29 | -0.09 | 0.29 | 1.05 | -0.39 |
| hV2v | 0.65 | -0.83 | 0.49 | -0.77 | -0.58 | 0.70 | 0.40 | -0.47 | -0.58 | -0.26 | -0.21 | 0.20 | -0.85 | 0.01 |
| hV3A | 0.02 | 2.15 | -1.09 | -0.68 | 1.18 | 1.43 | -1.46 | -0.86 | 0.59 | 1.76 | -0.56 | 0.71 | -0.92 | 0.75 |
| hV3d | 0.71 | -0.73 | -0.38 | -0.44 | -1.62 | -1.56 | -0.44 | -0.60 | -1.16 | 0.36 | 0.07 | 1.12 | -0.31 | -0.37 |
| hV3v | 0.97 | 0.01 | 0.03 | 0.07 | 0.77 | -0.70 | 0.25 | -0.24 | -0.90 | -0.87 | -0.24 | 0.84 | 0.24 | -0.08 |
| hV4v | -2.08 | 0.21 | -1.64 | -1.14 | 0.77 | 0.75 | -1.39 | -0.94 | -0.54 | -1.65 | -1.69 | -0.28 | -1.30 | -1.99 |

**Supplementary Table 8.** Z-score normalized receptor densities used for cluster analyses depicted in Figure 9. Note, that for each receptor type normalization was carried out together for macaque and human areas. Cells have been color-coded to facilitate identification of areas with higher (warm tones) and lower (cool tones) densities than the average for the respective receptor type.

|  | AMPA | kainate | NMDA | GABAᴀ | GABAᴃ | GABAᴀ/BZ | M₁ | M₂ | M₃ | α₁ | α₂ | 5-HT₁ᴀ | 5-HT₂ | D₁ |
| --- | --- | --- | --- | --- | --- | --- | --- | --- | --- | --- | --- | --- | --- | --- |
| mV1d | -0.30 | 0.16 | 0.85 | 0.57 | 1.70 | 1.55 | 0.74 | 1.39 | -0.14 | -1.07 | -0.03 | -1.44 | 1.88 | -0.52 |
| mV1v | 0.45 | 0.32 | 0.95 | 0.43 | 0.25 | 0.33 | 0.74 | 1.81 | -0.47 | -0.92 | 0.11 | -1.49 | 2.37 | -0.40 |
| mV2d | 0.65 | 1.27 | -0.31 | -0.36 | 1.15 | -0.27 | 1.03 | -0.55 | -1.08 | -0.62 | -0.91 | -0.79 | -0.54 | -1.08 |
| mV2v | -0.16 | 0.67 | -0.11 | -0.42 | -0.19 | 0.42 | 1.56 | -0.50 | -1.05 | -1.45 | -0.81 | -0.75 | -0.05 | -1.20 |
| mV3A | 1.78 | 0.24 | -0.50 | -0.90 | -0.52 | -1.24 | 0.79 | -1.04 | -0.82 | -0.16 | -0.95 | -0.05 | -0.21 | -0.79 |
| mV3d | 0.44 | -0.11 | -0.31 | -0.83 | -0.60 | -1.32 | 0.90 | -0.51 | -0.92 | -1.06 | -0.94 | -1.02 | -0.26 | -0.73 |
| mV3v | 1.07 | 1.61 | -0.43 | -0.88 | -1.37 | -0.54 | 1.08 | -0.72 | -0.93 | -0.73 | -0.94 | -0.28 | 0.55 | -1.60 |
| mV4v | -0.77 | 1.90 | -0.21 | -1.13 | -0.14 | 0.46 | 0.58 | -0.87 | -0.66 | 0.04 | -0.99 | -0.51 | 0.87 | -0.85 |
| hV1d | -0.42 | -1.25 | 1.60 | 2.15 | 0.63 | -0.85 | -0.61 | 1.59 | 1.00 | 0.77 | 2.02 | 0.02 | 0.13 | 1.24 |
| hV1v | -1.17 | -0.90 | 1.52 | 2.12 | -0.15 | -0.18 | -0.66 | 1.62 | 2.38 | 1.02 | 1.90 | -0.35 | -0.26 | 1.37 |
| hV2d | 0.13 | -0.56 | 0.39 | 0.41 | -1.15 | 0.58 | -0.78 | 0.15 | 1.09 | 0.98 | 0.59 | 0.99 | -0.06 | 0.74 |
| hV2v | 0.30 | -1.20 | 0.66 | -0.44 | -0.66 | 0.85 | -0.82 | -0.33 | 0.27 | 0.54 | 0.49 | 0.93 | -1.00 | 0.90 |
| hV3A | -0.38 | 0.38 | -1.46 | -0.33 | 1.24 | 1.64 | -1.34 | -0.70 | 1.26 | 2.15 | 0.16 | 1.27 | -1.03 | 1.19 |
| hV3d | 0.37 | -1.14 | -0.50 | -0.06 | -1.78 | -1.64 | -1.05 | -0.45 | -0.23 | 1.03 | 0.75 | 1.54 | -0.73 | 0.75 |
| hV3v | 0.64 | -0.75 | 0.06 | 0.52 | 0.80 | -0.70 | -0.86 | -0.10 | 0.00 | 0.05 | 0.45 | 1.35 | -0.46 | 0.87 |
| hV4v | -2.61 | -0.64 | -2.21 | -0.86 | 0.79 | 0.90 | -1.32 | -0.78 | 0.30 | -0.57 | -0.91 | 0.60 | -1.22 | 0.11 |
